# Post-COVID-19 Jaw Osteonecrosis: A Narrative Review

**DOI:** 10.3390/medicina62040641

**Published:** 2026-03-27

**Authors:** George Cătălin Alexandru, Loredana-Neli Gligor, Doina Chioran, Ciprian I. Roi, Mircea Riviș, Marius Octavian Pricop, Andrei Urîtu, Aliteia-Maria Pacnejer, Horațiu Cristian Manea, Tudor Rareş Olariu

**Affiliations:** 1 Doctoral School, “Victor Babes” University of Medicine and Pharmacy Timisoara, Eftimie Murgu Sq. No. 2, 300041 Timisoara, Romania; george.alexandru@umft.ro (G.C.A.); loredana.alexandru@umft.ro (L.-N.G.); 2 Center for Diagnosis and Study of Parasitic Diseases, Department of Infectious Disease, “Victor Babes” University of Medicine and Pharmacy Timisoara, Eftimie Murgu Sq. No. 2, 300041 Timisoara, Romania; rolariu@umft.ro; 3 Department of Pulmonology, Center for Research and Innovation in Precision Medicine of Respiratory Diseases, “Victor Babes” University of Medicine and Pharmacy Timisoara, Eftimie Murgu Sq. No. 2, 300041 Timisoara, Romania; 4 Department of Anesthesiology and Oral Surgery, “Victor Babes” University of Medicine and Pharmacy Timisoara, Eftimie Murgu Sq. No. 2, 300041 Timisoara, Romania; andrei.uritu@umft.ro; 5 Research Center of Dento-Alveolar Surgery, Anesthesia and Sedation in Dental Medicine, University Clinic of Anesthesiology and Oral Surgery, “Victor Babes” University of Medicine and Pharmacy Timisoara, Eftimie Murgu Sq. No. 2, 300041 Timisoara, Romania; rivis.mircea@umft.ro; 6 Department of Oral and Maxillofacial Surgery, “Victor Babes” University of Medicine and Pharmacy Timisoara, Eftimie Murgu Sq. No. 2, 300041 Timisoara, Romania; pricop.marius@umft.ro; 7 Department of Toxicology, “Victor Babes” University of Medicine and Pharmacy Timisoara, Eftimie Murgu Sq. No. 2, 300041 Timisoara, Romania; aliteia.pacnejer@umft.ro; 8 Research Centre for Pharmaco-Toxicological Evaluation, “Victor Babes” University of Medicine and Pharmacy Timisoara, Eftimie Murgu Sq. No. 2, 300041 Timisoara, Romania; 9 Faculty of Nursing, Department I—Nursing, University Clinic of Clinical Skills, “Victor Babes” University of Medicine and Pharmacy Timisoara, Eftimie Murgu Sq. No. 2, 300041 Timisoara, Romania; horatiu.manea@umft.ro; 10 Discipline of Parasitology, Department of Infectious Diseases, “Victor Babes” University of Medicine and Pharmacy Timisoara, Eftimie Murgu Sq. No. 2, 300041 Timisoara, Romania; 11 Clinical Laboratory, Municipal Clinical Emergency Hospital, 300254 Timisoara, Romania; 12 Patogen Preventia, 300124 Timisoara, Romania

**Keywords:** post-COVID-19, jaw osteonecrosis, corticosteroids, diabetes, mucormycosis

## Abstract

*Background and Objectives*: Osteonecrosis of the jaw (ONJ) occurring after infection with SARS-CoV-2 has emerged as an increasingly reported complication in the post-COVID-19 era. Post-COVID-19 osteonecrosis of the jaw (PC-ONJ) has been described in association with both COVID-19-associated mucormycosis (CAM) and non-fungal phenotypes. This narrative review aims to synthesize and critically analyze the available evidence regarding terminology and classification, epidemiology and risk factors, pathophysiological mechanisms, clinical and imaging characteristics, diagnostic challenges, and management strategies relevant to oral and maxillofacial surgery practice. *Materials and Methods*: An extensive literature search was conducted in the PubMed/MEDLINE, Scopus, Web of Science, ScienceDirect, and Google Scholar databases. The search targeted peer-reviewed publications published between 2020 and 2025, reflecting the post-pandemic emergence of this clinical spectrum. Original studies, systematic and narrative reviews, multicenter case series, consensus guidelines, and well-documented case reports were considered. *Results*: Available data, largely derived from case reports and small series, demonstrate a predominance of maxillary involvement and frequent association with diabetes mellitus and systemic corticosteroid therapy. Proposed mechanisms include COVID-19-associated endothelial dysfunction, microvascular thrombosis, immune dysregulation, metabolic imbalance, and treatment-related effects. Clinically, patients may present with persistent orofacial pain, tooth mobility, exposed or probeable bone, and frequent sinonasal extension, with symptoms sometimes preceding bone exposure. Diagnostic challenges arise from the overlap with medication-related osteonecrosis of the jaw (MRONJ), osteoradionecrosis (ORN), and chronic osteomyelitis. Imaging is essential for assessing disease extent but remains insufficient for etiologic differentiation, making histopathological examination and targeted microbiological investigations necessary, particularly to exclude invasive fungal infection. *Conclusions*: Management must be etiology-driven. CAM requires urgent antifungal therapy combined with surgical debridement, whereas non-fungal forms are generally managed with conservative surgery and appropriate antimicrobial stewardship. Standardized diagnostic criteria and prospective multicenter studies are needed to reduce nosological ambiguity and optimize clinical decision-making in this emerging post-viral condition.

## 1. Introduction

Since its emergence in late 2019, infection with severe acute respiratory syndrome coronavirus 2 (SARS-CoV-2) has been recognized as a systemic disease with multisystem involvement extending far beyond the respiratory tract. In addition to acute viral injury, COVID-19 is now understood to induce a complex post-infectious state characterized by endothelial dysfunction, immune dysregulation, hypercoagulability, and metabolic derangements, which together contribute to a wide spectrum of delayed complications affecting multiple organs [1,2]. As the number of COVID-19 survivors has increased globally, attention has progressively shifted toward the identification and characterization of post-acute sequelae, including previously rare or poorly defined manifestations involving the oral and maxillofacial regions [3,4].

Among these emerging complications, osteonecrotic and osteomyelitic involvement of the jaws occurring after COVID-19 has gained relevance. Since mid-2020, an increasing number of cases describing maxillary or mandibular bone necrosis developing weeks to months after SARS-CoV-2 infection have been reported worldwide, initially in association with CAM and later also in the apparent absence of invasive fungal disease or conventional osteonecrosis risk factors [5,6]. These lesions have been variably termed COVID-19-related osteonecrosis of the jaw (CRONJ), PC-ONJ, post-COVID-19-related osteonecrosis (PC-RONJ), or simply post-COVID-19 osteomyelitis, reflecting the lack of nosological consensus and the heterogeneity of the reported phenotypes.

Clinically, post-COVID-19 jaw lesions present with overlapping features that include persistent orofacial pain, sudden tooth mobility, exposed or probing bone, palatal or alveolar ulceration, fistula formation, and frequent maxillary sinus involvement. Importantly, symptoms may precede bone exposure by weeks or months, complicating early recognition and frequently leading to misclassification as odontogenic infection, chronic osteomyelitis, MRONJ, or ORN [5,7]. Radiological findings further contribute to diagnostic ambiguity, as osteolysis, sequestration, and cortical destruction are not pathognomonic and may be shared across ischemic, infectious, and medication- or radiation-related processes [8].

From a pathogenic standpoint, accumulating evidence suggests that PC-ONJ represents a multifactorial spectrum rather than a single disease entity. Proposed mechanisms include COVID-19-associated coagulopathy with microvascular thrombosis, endothelial injury mediated by inflammatory cytokines and ACE2 dysregulation, immune suppression and metabolic imbalance favoring secondary infection, and treatment-related effects, particularly systemic corticosteroid exposure and steroid-induced hyperglycemia [1,7,9]. In some patients, invasive fungal infection, most notably mucormycosis, acts as the dominant driver of rapid bone necrosis, whereas in others, an apparently aseptic, ischemic mechanism predominates, with negative fungal and bacterial investigations [7,10].

Although the literature on this topic is expanding, important gaps remain. Most available data derive from isolated case reports and small series, frequently without uniform diagnostic definitions, comprehensive microbiological work-up, or adequate follow-up. The interchangeable use of the terms “osteomyelitis” and “osteonecrosis,” inconsistent exclusion of CAM, and limited integration of systemic risk factors have further contributed to conceptual confusion. Consequently, clinicians face real diagnostic and therapeutic dilemmas, with a risk of both overdiagnosis of invasive fungal disease and underrecognition of ischemic post-viral bone injury [5,11].

This review offers a novel contribution by presenting a comprehensive, etiology-driven framework for understanding post-COVID-19 jaw osteonecrosis. Instead of considering PC-ONJ as a singular condition, this paper offers a critical synthesis of existing research across infectious (CAM-related), non-fungal ischemic, and mixed phenotypes. It addresses diagnostic challenges, nosological uncertainties, and the overlapping clinical features. Furthermore, this paper seeks to bridge current research deficiencies by synthesizing epidemiological data, pathophysiological processes, imaging results, histopathological features, and treatment strategies into a unified clinical framework, highlighting the importance of rigorous exclusion criteria and collaborative assessment.

The primary objective is to provide a thorough examination supported by evidence of post-COVID-19 jaw osteonecrosis. It aims to clarify terminology, underlying mechanisms, diagnostic protocols and challenges, differential diagnosis, management strategies, and prognostic considerations relevant to oral and maxillofacial surgery practice. Furthermore, it seeks to propose a structured conceptual framework that facilitates precise classification, prompt intervention, and informed clinical judgment in this novel and intricate post-viral ailment.

## 2. Materials and Methods

An extensive search was conducted on the literature available on PubMed/MEDLINE, Scopus, Web of Science, ScienceDirect, and Google Scholar databases. The search strategy targeted peer-reviewed articles published between 2020 and 2025, reflecting the post-pandemic emergence of this clinical spectrum. Earlier literature was selectively included when necessary to contextualize established entities such as MRONJ, ORN, chronic osteomyelitis, and invasive fungal infections of the maxillofacial region. Search terms and combinations included, but were not limited to: COVID-19, SARS-CoV-2, osteonecrosis of the jaw, post-COVID-19 jaw necrosis, COVID-19-associated mucormycosis, rhino-maxillary mucormycosis, jaw osteomyelitis, avascular necrosis, endotheliopathy, and microthrombosis. Publications written in English were considered eligible. The initial search across PubMed/MEDLINE, Scopus, Web of Science, ScienceDirect, and Google Scholar retrieved 300 records. After the search results were identified, duplicate publications retrieved from multiple databases were removed. After removing duplicates and screening titles, abstracts, and full texts according to the eligibility criteria, 60 publications directly relevant to PC-ONJ and related maxillofacial complications were included in the qualitative synthesis, while additional references were used to provide background on pathophysiology, terminology, differential diagnosis, and management.

Original studies, systematic and narrative reviews, multicenter case series, as well as consensus guidelines and position papers developed by authorities in the fields of oral and maxillofacial surgery, infectious diseases, radiology, and pathology were primarily considered. Well-documented case series were also included when they provided detailed clinical, imaging, histopathological, or therapeutic information, particularly regarding non-fungal PC-ONJ phenotypes, for which higher-level evidence is still limited. Individual case reports were considered selectively, only when they provided examples of novel mechanisms, diagnostic difficulties, or atypical presentations, supported by a clear methodological description.

Although the paper is not a systematic review per se, the process of identifying and selecting the literature followed adapted PRISMA principles, and eligibility criteria were established in advance to ensure transparency and consistency. Publications were included that addressed maxillary osteonecrosis or osteomyelitis occurring during or after SARS-CoV-2 infection; that reported clinical, imaging, histopathological, microbiological, or therapeutic data relevant to PC-ONJ or CAM-associated pathologies; or that discussed pathophysiological mechanisms linking COVID-19, systemic treatments (e.g., corticosteroids) and maxillary bone necrosis. Abstracts and conference communications without sufficient clinical or methodological details, as well as studies not relevant to the maxillofacial field, were excluded. Also, papers that focused only on non-COVID-19-related osteonecrosis of the jaw were excluded unless they provided essential background information necessary to contextualize the pathophysiology, terminology, or management of post-COVID-19 jaw necrosis.

Data extraction and analysis were performed qualitatively, with a focus on identifying recurrent clinical patterns, common risk factors, diagnostic overlaps, and differences between post-COVID-19 osteonecrosis associated with CAM and non-fungal forms. Special attention was paid to the diagnostic stages, including the role of imaging, biopsy, histopathological examination, and targeted microbiological investigations, as well as the therapeutic decision-making process and multidisciplinary case management.

## 3. Terminology and Classification of Jaw Osteonecrosis

### 3.1. Evolution of Terminology

Jaw osteonecrosis encompasses a spectrum of conditions characterized by the death of bone tissue in the mandible or maxilla due to failure of normal bone homeostasis rather than a single mechanism. Historically, terminology developed around assumptions about etiology, especially vascular insufficiency. As a consequence, it resulted in a variety of overlapping and sometimes misleading labels that reflected presumed mechanisms rather than demonstrated pathophysiology. Early terms, such as “aseptic necrosis”, were introduced to distinguish non-infectious bone death from septic osteomyelitis, emphasizing the absence of infection, rather than the underlying mechanism. Later, the labels “avascular necrosis” and “ischemic necrosis” shifted the focus explicitly to loss of blood supply as the presumed primary cause of bone death despite limited direct evidence in many non-traumatic cases. Although vascular insufficiency is one contributor to osseous injury, contemporary evidence demonstrates that jaw osteonecrosis is multifactorial, involving impaired bone turnover, direct cellular toxicity, altered angiogenesis, inflammation, systemic disease, and infection [12,13,14].

By the 1990s, a consensus group (ARCO—Association Research Circulation Osseous) adopted “osteonecrosis” as the preferred term. Nevertheless, earlier, narrower expressions like “avascular necrosis” remain entrenched in the literature [15].

Recent data increasingly support the view that non-traumatic osteonecrosis is not exclusively vascular. In the jaws, bone death reflects disrupted bone homeostasis that can be driven by vascular compromise or by non-vascular mechanisms. Because terms like “avascular” or “ischemic” necrosis point to a single pathway, they risk oversimplifying a multifactorial disease process and may bias diagnostic and therapeutic thinking toward revascularization alone [12].

For these reasons, contemporary literature increasingly recommends abandoning “avascular necrosis” for the jaws and using “osteonecrosis” as the umbrella term, with precise modifiers that reflect etiology [13].

### 3.2. Current Consensus Terminology for the Jaws

For jaw bones, several specific entities are now recognized. ONJ serves as an umbrella term that describes bone death in the maxilla or mandible of diverse etiologies. From a classificatory perspective (Table 1), ONJ can be subdivided etiologically into medicated-related, radion-related, traumatic, non-traumatic systemic or infectious, and spontaneous or idiopathic forms [13].

MRONJ represents the current consensus terminology for drug-induced ONJ. This term has replaced earlier nomenclature, including bisphosphonate-related osteonecrosis of the jaw (BRONJ), bisphosphonate-related MRONJ (BRMRONJ), and similar variants, in order to encompass a broader spectrum of causative agents. MRONJ encompasses osteonecrosis associated with antiresorptive medications, such as bisphosphonates and denosumab, as well as antiangiogenic and targeted therapies, including bevacizumab, sunitinib, and other vascular endothelial growth factor (VEGF) or tyrosine kinase inhibitors [14,16]. Clinically, MRONJ is defined by current or previous exposure to these agents, the presence of exposed bone or bone that can be probed through a fistula in the maxillofacial region persisting for more than eight weeks, and the absence of prior radiation therapy to the jaws or evidence of metastatic disease involving the jaw bones. Some European expert groups have suggested shortening the eight-week observation period to avoid delays in diagnosis and treatment [13,16].

ORN, also referred to as osteoradionecrosis of the jaws, denotes necrosis of previously irradiated jaw bone, most commonly occurring in patients treated for head and neck malignancies. ORN has traditionally been defined as exposed irradiated bone that fails to heal for at least three months in the absence of persistent or recurrent tumor. Its pathogenesis is characterized by chronic hypoxia, hypovascularity, and hypocellularity, accompanied by radiation-induced fibrosis, all of which impair normal bone remodeling and wound healing [13].

Traumatic ONJ arises following thermal, mechanical, or chemical injury to bone. This includes maxillofacial fractures or osteotomies associated with vascular disruption, as well as iatrogenic injuries (i.e., aggressive curettage, prolonged pressure from endotracheal tubes, inappropriate use of devitalizing agents, sodium hypochlorite accidents, or exposure to strong acids). On the other hand, non-traumatic ONJ occurs in the absence of medication exposure or radiation therapy and is typically associated with systemic or infectious conditions. Infectious causes include osteomyelitis, noma, tuberculosis, syphilis, actinomycosis, fungal infections such as mucormycosis and aspergillosis, and herpes zoster-associated lesions. Systemic diseases implicated in this category include diabetes mellitus, coagulation disorders such as thrombophilia, hypofibrinolysis, and disseminated intravascular coagulation, hemoglobinopathies, autoimmune diseases, immunodeficiency states, and other vascular disorders. In addition, bone infiltration by primary or metastatic malignancies and tissue destruction related to narcotic use, including intranasal cocaine and desomorphine (“krokodil”), may result in osteonecrosis [13,17].

Spontaneous or idiopathic ONJ refers to exposed bone lesions that develop without any identifiable precipitating medication, radiation exposure, systemic disease, or recognizable trauma. These lesions, often described in the literature as “oral ulceration with bone sequestration” or idiopathic exposed bone lesions, can be linked to bony prominences like tori or exostoses [13].

Historically, the term “phossy jaw”, or phosphorus necrosis, describes ONJ seen in match factory workers who were exposed to white phosphorus. This condition appeared before the use of modern antiresorptive and cancer treatments [18].

Overall, contemporary consensus favors the use of the term “ONJ” combined with an etiologic modifier, such as MRONJ, ORN, traumatic ONJ, infection-related ONJ, or idiopathic ONJ.

### 3.3. Etiologic Classification of Jaw Osteonecrosis

Based on current understanding, jaw osteonecrosis can be logically classified according to etiology.

MRONJ is associated with antiresorptive agents, including oral and intravenous bisphosphonates and denosumab, as well as antiangiogenic and targeted therapies such as bevacizumab, sunitinib, other tyrosine kinase inhibitors, and mTOR inhibitors. The pathogenesis of MRONJ is multifactorial and includes profound suppression of bone turnover, impaired coupling between bone resorption and angiogenesis, direct soft-tissue toxicity, and secondary infections [14,19,20].

ORN occurs post-radiation therapy to the jaws. Typically, the risk increases substantially at cumulative doses above approximately 50–60 Gy or higher, although cases have been reported at lower doses. Its pathophysiology involves radiation-induced endarteritis, tissue hypoxia, fibrosis, reduced cellularity, and impaired healing capacity [21].

Traumatic osteonecrosis results from major trauma such as fractures, osteotomies, or surgical procedures, which leads to vascular compromise, as well as iatrogenic, chemical, or thermal injuries, including devitalizing pastes, sodium hypochlorite injection, excessive heat during drilling, or acid burns [13].

Osteonecrosis of infectious origin can be met in the context of chronic osteomyelitis with bone sequestration, severe infections (noma, tuberculosis, syphilis, actinomycosis, fungal osteomyelitis), or in lesions associated with herpes zoster infection. In such situations, the necrotic bone is generally exposed or visible, and the clinical picture is accompanied by obvious signs of severe soft tissue damage [13].

Osteonecrosis linked with systemic disease is associated with conditions that impair vascular supply, immunity, or metabolism, including poorly controlled diabetes mellitus, coagulation disorders, hematologic diseases, autoimmune conditions, and other vascular pathologies [13,14,18,22].

Neoplasm-associated osteonecrosis usually involves the destruction and necrosis of bone. It results from primary or metastatic malignancies infiltrating the jawbones. Before making a diagnosis of MRONJ, these conditions must be excluded [23,24].

Bone necrosis can also be induced due to drug and lifestyle factors, and results in soft-tissue destruction that leads to osteonecrosis associated with substances such as cocaine and desomorphine, which induce ischemia and chemical tissue damage [17,25].

Finally, idiopathic or spontaneous osteonecrosis is characterized by small exposed bony sequestra without an identifiable etiologic factor. Frequently, it occurs on the lingual surfaces of the mandible or over bony prominences [26].

This etiologic classification complements, rather than replaces, clinical staging systems, which are primarily based on clinical presentation, symptom severity, and disease extent.

### 3.4. Clinical Staging and Classification Systems

#### 3.4.1. MRONJ: AAOMS Staging

The American Association of Oral and Maxillofacial Surgeons (AAOMS) proposed a staging system in 2006 that was subsequently updated in 2009, 2014, and 2022. It is the most widely used classification for MRONJ, applying to patients with relevant exposure to antiresorptive or antiangiogenic agents and no history of jaw irradiation or metastatic disease [16,24].

Patients categorized at risk have a history of exposure to certain medications but show no clinical evidence of necrotic bone.

Stage 0 represents a non-exposed variant characterized by the absence of visible bone, but with symptoms that are not specific or radiographic changes such as pain, tooth loosening, sinus symptoms, sclerosis, periodontal ligament widening, or non-healing extraction sockets. Some oncology societies consider this stage a warning category rather than definitive MRONJ. Nonetheless, a proportion of cases may progress.

Stage 1 is marked by exposed and necrotic bone or a fistula probing to bone without the presence of pain, infection, or erythema.

In Stage 2, the necrotic bone is apparent or a fistula probe shows bone, and there is pain, redness, and/or pus coming out of the wound, which is a sign of infection.

Stage 3 is the last stage of the disease. It is marked by exposed necrotic bone, pain, infection, and at least one complication, such as a pathologic fracture, an extraoral fistula, an oroantral or oronasal communication, or radiographic osteolysis that goes to the bottom of the mandible or the floor of the maxillary sinus [16,18,24,27,28].

This staging system is based on severity and directly informs management strategies, which range from conservative measures in early stages to surgical intervention in advanced disease.

#### 3.4.2. ORN Classifications

Unlike MRONJ, ORN does not benefit from a single, widely accepted classification system. Over time, several classification schemes have been proposed, based on the onset and progression of the disease [29]. These include the Morton and Simpson (1986) classification, an early model based on clinical progression, which divides ORN into four categories. Thus, the mild form includes limited ulcerations with bone exposure, which tend to resolve spontaneously within a few months; the moderate form is characterized by bone exposure accompanied by sequestration, usually responding to conservative treatment within six to twelve months; and the severe form involves extensive bone exposure, the appearance of fistulas, possible pathological fractures, and progressive deterioration that often requires surgical intervention [13].

Kagan and Schwartz (2002) system is another widely cited classification, in which the extent of bone involvement and the degree of soft-tissue damage are emphasized. In Stage I, disease involvement is superficial and restricted to the cortical bone, with only mild soft-tissue ulceration, and management is usually conservative. In Stage II there is a limited extension of the disease into the medullary bone. Stage II is subdivided in Stage IIa, characterized by minimal soft-tissue ulceration, and Stage IIb, associated with significant soft-tissue necrosis or the development of an orocutaneous fistula. Usually, management strategies include conservative approaches or less invasive surgical procedures. Stage III is characterized by complete bone necrosis and may present with pathological fractures, the development of fistulas, or necrosis of the overlying integument. Furthermore, Stage III is subdivided into IIIa and IIIb. Stage IIIa is characterized by limited soft-tissue involvement, whereas Stage IIIb involves extensive soft-tissue necrosis or related complications. Cases classified as IIIb often require aggressive surgical resection followed by reconstructive procedures [13]. In the 2020s, recent consensus efforts have proposed updated definitions of ORN by including clinical findings, imaging features, and radiation history. However, these approaches continue to evolve and may vary among guidelines [29].

**Table 1:** Terminology, etiologic classification, and clinical staging systems of jaw osteonecrosis.

Section	Classification	Definition and Scope	Key Characteristics	Ref.
**Terminology**	ONJ	Jaw osteonecrosis encompasses a spectrum of conditions characterized by death of bone tissue in the mandible or maxilla due to failure of normal bone homeostasis rather than a single pathogenic mechanism.	Multifactorial process involving impaired bone turnover, direct cellular toxicity, altered angiogenesis, inflammation, infection, and systemic disease; vascular insufficiency may contribute but is not exclusive.	[12,13,14]
**Historical terminology**	“Aseptic necrosis”; “avascular necrosis”; “ischemic necrosis”	Earlier terms developed to distinguish noninfectious bone death or to imply vascular insufficiency as the primary mechanism of necrosis.	These labels reflect presumed mechanisms rather than demonstrated pathophysiology and may oversimplify a multifactorial disease process, particularly in non-traumatic jaw lesions.	[12,15]
**Consensus terminology adoption**	Osteonecrosis (preferred umbrella term)	By the 1990s, consensus efforts in the broader non-traumatic osteonecrosis literature, including ARCO, adopted “osteonecrosis” as the preferred term.	Narrower expressions such as “avascular necrosis” remain entrenched in the literature despite limited applicability to multifactorial jaw disease.	[15]
**Medication-related entity**	MRONJ	ONJ associated with exposure to antiresorptive, antiangiogenic, or targeted therapies.	Defined by current or previous exposure to these agents, exposed bone or bone probeable through a fistula persisting for more than 8 weeks, and absence of prior radiation therapy to the jaws or metastatic jaw disease; some European expert groups propose shortening the observation period to avoid diagnostic delay.	[13,14,16]
**Radiation-related entity**	ORN	Necrosis of jaw bone previously exposed to therapeutic ionizing radiation, most commonly in patients treated for head and neck malignancies.	Traditionally defined as exposed irradiated bone failing to heal for at least three months in the absence of persistent or recurrent tumor; pathogenesis includes hypoxia, hypovascularity, hypocellularity, and radiation-induced fibrosis.	[13]
**Traumatic entity**	Traumatic ONJ	Osteonecrosis arising from thermal, mechanical, or chemical injury to jaw bone.	Includes maxillofacial fractures, osteotomies with vascular disruption, and iatrogenic injuries such as aggressive curettage, devitalizing agents, sodium hypochlorite accidents, excessive heat during drilling, or exposure to strong acids.	[13]
**Infection-related entity**	Infection-related ONJ	Osteonecrosis occurring secondary to infectious processes affecting the jaw bones.	Includes chronic osteomyelitis with sequestration, noma, tuberculosis, syphilis, actinomycosis, fungal infections (e.g., mucormycosis, aspergillosis), and herpes zoster-associated lesions; typically accompanied by significant soft-tissue infection.	[13]
**Systemic disease-related entity**	Systemic disease-related ONJ	Osteonecrosis associated with systemic conditions that impair vascular supply, immunity, or metabolic homeostasis.	Includes diabetes mellitus, coagulation disorders (thrombophilia, hypofibrinolysis, disseminated intravascular coagulation), hemoglobinopathies, autoimmune diseases, immunodeficiency states, and other vascular disorders.	[13,14,18]
**Neoplasm-associated entity**	Neoplasm-associated ONJ	Bone destruction and necrosis secondary to infiltration by primary or metastatic malignancies involving the jaw bones.	Must be excluded before establishing a diagnosis of MRONJ.	[23,24]
**Drug- and lifestyle-related lesions**	Substance-associated osteonecrosis	Osteonecrosis resulting from ischemic and chemical tissue destruction associated with substance use.	Includes lesions associated with intranasal cocaine and desomorphine (“krokodil”), which induce severe soft-tissue damage and secondary bone necrosis.	[13,17,25]
**Idiopathic entity**	Idiopathic/spontaneous ONJ	Exposed necrotic bone lesions developing without identifiable medication exposure, radiation therapy, systemic disease, infection, or recognizable trauma.	Often described as oral ulceration with bone sequestration; frequently occurs over bony prominences such as tori or exostoses, particularly on the lingual surface of the mandible.	[13,26]
**Historical occupational entity**	“Phossy jaw” (phosphorus necrosis)	ONJ observed in match factory workers exposed to white phosphorus.	An occupationally acquired condition characterized by progressive jawbone necrosis with sequestration and secondary infection following chronic exposure to white phosphorus.	[18]
**MRONJ clinical staging**	AAOMS staging system	Severity-based clinical staging system for MRONJ.	“At risk” category; Stage 0 (non-exposed variant with symptoms or radiographic changes); Stage 1 (exposed/probeable bone without infection); Stage 2 (exposed/probeable bone with pain and infection); Stage 3 (advanced disease with complications such as fracture, fistula, or extensive osteolysis).	[16,18,27]
**ORN classification systems**	Multiple ORN classifications	ORN lacks a single universally accepted staging system.	Includes progression-based systems (Morton and Simpson), extent-based systems (Kagan and Schwartz), and evolving consensus approaches integrating clinical findings, imaging features, and radiation history; non-exposed variants are increasingly recognized.	[13,28,29]

Abbreviations: ARCO, Association Research Circulation Osseous; AAOMS, American Association of Oral and Maxillofacial Surgeons. The listed categories may overlap in clinical practice and should be interpreted in the context of the patient’s history, risk factors, imaging findings, and histopathological evaluation.

## 4. Epidemiology and Risk Factors of Post-COVID-19 Osteonecrosis of the Jaw

Since mid-2020, over two hundred cases of COVID-19-related jaw osteonecrosis have been documented in the literature. A recent systematic review described 42 studies, in which 201 cases of COVID-associated osteomyelitis/osteonecrosis of the jaw (COMJ) were reported, indicating that this complication, while still relatively uncommon, has been observed worldwide. The median age of patients is around 50–55 years, but the range is broad (cases from adolescents to the elderly). Males have outnumbered females by about 3:1 among the cases. This male predominance in COMJ mirrors the male bias in severe COVID-19 outcomes, possibly because men have higher rates of risk factors like uncontrolled diabetes and are more susceptible to severe inflammatory responses. Indeed, in the compiled case data, diabetes mellitus was present in about 65% of post-COVID-19 jaw necrosis patients, underscoring diabetes as a major comorbidity [5,30].

In terms of anatomical distribution, the maxilla (upper jaw) has been affected far more often than the mandible in PC-ONJ cases. Approximately 90% of reported cases involve the maxilla (often with sinus involvement), whereas the mandible is involved in under 10%. This is atypical, since in non-COVID-19 osteomyelitis, the mandible is usually more frequently involved (due to its poorer blood supply). The reversal in PC-ONJ likely reflects the predilection of COVID-19-related fungal infections (like mucormycosis) for the sinonasal region, which then spread to the maxilla. It may also be that the maxillary bone is uniquely susceptible to COVID-19-related microangiopathic damage due to its richer vascular network (paradoxically providing more targets for thrombosis and fungal seeding) [5,31,32,33].

### 4.1. Key Risk Factors

#### 4.1.1. Corticosteroid Therapy

The majority of PC-ONJ patients had received high-dose corticosteroids during their COVID-19 illness. In the systematic review, 58.5% had corticosteroid treatment for COVID-19 [5]. Steroids are a double-edged sword: while life-saving in severe COVID-19 (to curb hyperinflammation), they predispose to infection and impair bone healing. Corticosteroids suppress immune responses and can lead to secondary fungal or bacterial infections, a clear risk for osteomyelitis. They also induce hyperglycemia; many COVID-19 patients on steroids become transiently diabetic or experience worsened glycemic control [34,35,36,37]. Reports from India indicate that systemic corticosteroid therapy was common among patients with COVID-associated mucormycosis, affecting approximately 87% of cases, and roughly one in five patients received regimens exceeding the recommended dose or duration [38]. Steroid-induced diabetes and lymphopenia synergistically increase susceptibility to mucormycosis and other opportunists. Moreover, corticosteroids themselves have been linked to avascular necrosis (e.g., osteonecrosis of the femoral head is a known complication of high-dose steroid use). Thus, steroid therapy for COVID-19 is viewed as a pivotal risk factor for PC-ONJ development, either by fostering infection or by directly causing osteonecrosis through ischemic mechanisms [38,39].

#### 4.1.2. Diabetes Mellitus

As noted, around two-thirds of PC-ONJ patients have diabetes. In the context of COVID-19, diabetes is a well-known risk factor for severe disease and was highly prevalent among those who developed secondary infections like mucormycosis. Uncontrolled hyperglycemia impairs neutrophil and macrophage function, reduces vascular endothelial health, and delays wound healing. In one analysis of COVID-associated mucormycosis in India, over 65% of patients had uncontrolled diabetes, making it the single most significant risk factor. Diabetic ketoacidosis in particular creates an acidic, glucose-rich environment in which Mucor fungi thrive (they have ketone reductase that enables growth in high glucose/acid conditions). Therefore, diabetic patients recovering from COVID-19 are at markedly elevated risk for jaw osteonecrosis, especially the infectious type. Good glycemic control has been emphasized as a key preventive measure [5,40,41,42].

#### 4.1.3. Severity of COVID-19 and Hospitalization

Patients who had severe COVID-19 (often requiring hospitalization or ICU care) form a large subset of PC-ONJ cases. In the 201-case review, 41.5% had been hospitalized for COVID-19. Severe illness often correlates with factors that can damage bone health: systemic inflammation, prolonged immobilization, high inflammatory cytokines, and aggressive treatments (like mechanical ventilation and high-flow oxygen that might predispose to sinus infections). Prolonged hospital stays can also mean higher exposure to broad-spectrum antibiotics and invasive devices, which increase infection risk. However, it is notable that not all PC-ONJ patients had severe acute COVID; some were managed at home yet later developed jaw necrosis, indicating that even moderate COVID-19 illness can precipitate this complication when other risk factors co-exist [5].

#### 4.1.4. Immunosuppression

Beyond steroids and diabetes, any condition causing immunosuppression in a COVID-19 patient raises the risk. Cancer patients, transplant recipients, or those on immunomodulatory treatments (e.g., tocilizumab for COVID-19 cytokine storm) are more vulnerable. For example, the use of anti-IL6 therapy like tocilizumab, though not as common as steroids, has been reported and could contribute to impaired immune control of fungal organisms. In one center, virtually all post-COVID-19 mucormycosis patients had some form of immunosuppressive therapy prior to onset. The general principle is that COVID-19 plus an immune-weakening factor creates an environment where normally rare infections (like mucor) can take hold and where bone-healing capacity is diminished [31,33,41,43,44].

#### 4.1.5. Local Factors: Dental Trauma and Extractions

Similar to classic ONJ, local trauma in the oral cavity has been noticed as a precipitant in many cases of PC-ONJ. In an Indian series of 13 patients, 11 had a recent tooth extraction or poorly fitting dentures that likely initiated mucosal breakdown. Dental extractions in a patient recovering from COVID-19 (especially if on steroids or with undiagnosed diabetes) can act as the “provoking event” that exposes bone and seeds to infection. Thus, a provocative dental procedure is considered part of a possible “triad”, along with COVID-induced coagulopathy and steroid use, that sets the stage for jaw osteonecrosis. Clinicians now advise that elective dental extractions in recently COVID-19-recovered patients be approached with caution, ensuring risk factors are mitigated (i.e., good glycemic control) to prevent this complication [5,6].

#### 4.1.6. Demographic Patterns

**Figure 1:**
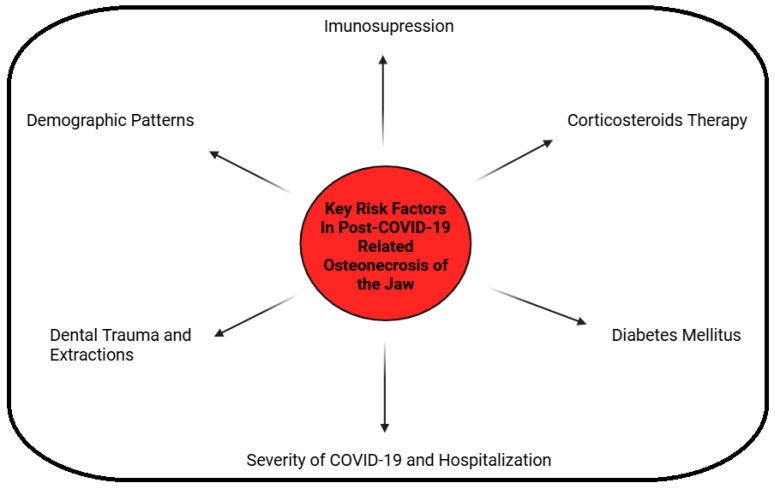
Image representing key risk factors associated with Post-COVID-19-related ONJ. The scheme was made using Biorender.com (accessed on 18 January 2026; https://www.biorender.com/).

The age range of reported PC-ONJ spans young to old, but many cases cluster in middle-aged and older adults (40s–60s). This likely reflects the age distribution of severe COVID-19 (which is rarer in children) and the fact that risk factors (Figure 1) like diabetes are more common in older adults. Nevertheless, pediatric cases have occurred: for example, an 8-year-old boy was reported among the 13-case series in India, showing that even young individuals can develop post-COVID-19 jaw necrosis if other factors align. As mentioned, men are more frequently affected than women. No strong data exist yet on ethnic predisposition (apart from regional differences discussed below), but it has been hypothesized that populations with a high prevalence of metabolic syndrome (central obesity, diabetes) and certain genetic prothrombotic tendencies might be more at risk for PC-ONJ. This remains to be studied [5,6,41,45].

So far, the available literature does not support SARS-CoV-2 infection as an independent etiological factor for jaw osteonecrosis in the absence of other predisposing conditions. Most reported PC-ONJ cases occur in the presence of other risk factors such as diabetes mellitus, systemic corticosteroid therapy, immunosuppression, or local triggering events (e.g., dental extraction). Current evidence suggests that SARS-CoV-2 infection acts primarily as a pathophysiological amplifier, which promotes endothelial dysfunction, microvascular thrombosis, immune dysregulation, and metabolic disturbances that lower the threshold for ischemic or infectious bone injury. In this context, COVID-19 should be interpreted as a contextual or synergistic factor that interacts with underlying comorbidities and treatment exposures rather than as a sole causal agent.

## 5. Pathophysiological Mechanisms Linking COVID-19 to Jaw Necrosis

The development of post-COVID-19 ONJ is likely multifactorial. It comes from an interplay of the virus’s effects, the immune-inflammatory response, and treatments used.

### 5.1. COVID-19 Coagulopathy and Microthrombosis

A hallmark of moderate to severe COVID-19 is the development of coagulopathy (CAC), which is a distinct prothrombotic state characterized by systemic inflammation, endothelial dysfunction, and dysregulated coagulation. Unlike classical disseminated intravascular coagulation, CAC is driven primarily by endothelial activation and immunothrombosis, resulting in widespread microvascular thrombosis [1,2].

**Figure 2:**
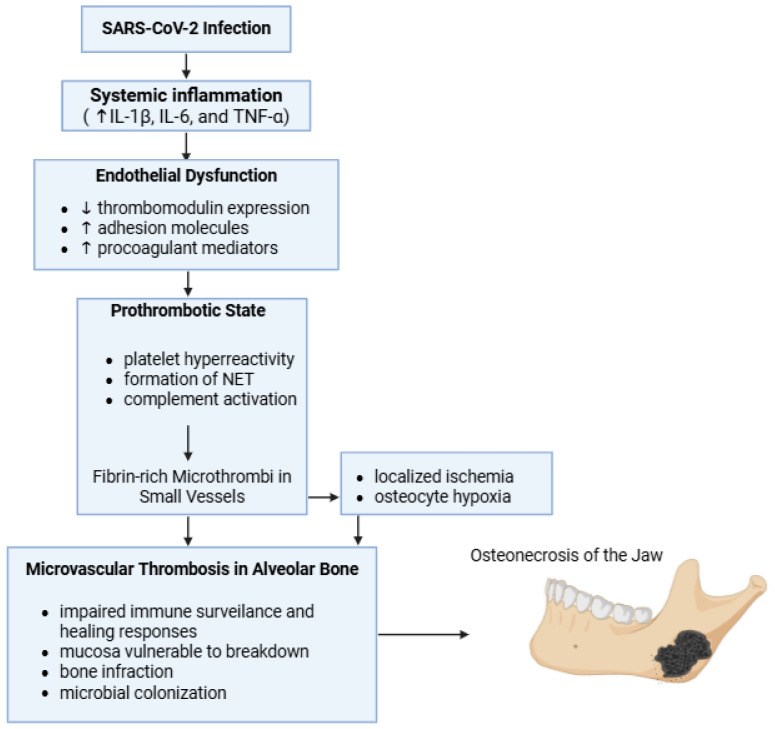
Representative scheme illustrating the proposed mechanism of post-COVID-19-related ONJ mediated by coagulopathy and microthrombosis. The scheme was made using Biorender.com (Accessed on 18 January 2026; https://www.biorender.com/).

ARS-CoV-2 infection causes the release of proinflammatory cytokines such as IL-1β, IL-6, and TNF-α. Because of that, the endothelium partially loses its anticoagulant properties, a phenomenon reflected by reduced thrombomodulin expression and increased adhesion molecules and procoagulant mediators (Figure 2). At the same time, activated monocytes and endothelial cells amplify tissue factor expression, which triggers the extrinsic pathway of coagulation and promotes excessive thrombin production. The prothrombotic state is further accentuated by platelet hyperactivity, the formation of neutrophil extracellular traps (NETs), and the activation of the complement system, processes that contribute to the development of fibrin-rich microthrombi in small vessels [46,47].

Multiple autopsy studies from patients who died of COVID-19 have confirmed the presence of microthrombi in multiple organ systems, supporting the systemic nature of this microvascular injury [2].

The maxillofacial skeleton can be significantly influenced by these mechanisms. The alveolar bone of the jaws is irrigated by a rich network of small intraosseous vessels, which maintain tissue with intense metabolic activity and a high capacity for renewal, due to the continuous remodeling involved in mastication and periodontal function. When these terminal microvessels are obstructed by thrombi, even locally, ischemia and hypoxia may occur in the osteocytes, which favors the onset of bone infarction. Initially, these microinfarcts may not produce obvious clinical manifestations, but they affect bone vitality and reduce its healing potential [7].

After the onset of ischemic injury, the affected bone becomes more vulnerable to secondary damage. Decreased perfusion compromises local immune defense mechanisms and healing processes, causing the covering mucosa to deteriorate more easily under the action of minor factors such as mechanical stress, periodontal inflammation, or routine dental procedures. Once exposed, devitalized bone can be colonized by oral microorganisms, resulting in polymicrobial cultures that are not, however, the primary cause of necrosis [7].

Several clinical observations support this ischemic–thrombotic mechanism. PC-ONJ has been reported predominantly in patients with a recent history of COVID-19, often following systemic corticosteroid therapy, and frequently in the absence of traditional osteonecrosis risk factors such as antiresorptive medications or radiotherapy. Corticosteroids may act synergistically with CAC by exacerbating endothelial dysfunction, promoting lipid embolism, and impairing bone microcirculation, thereby lowering the threshold for ischemic injury. In this context, COVID-19-related microvascular dysfunction may establish a vulnerable baseline state in which relatively minor local triggers precipitate overt osteonecrosis [6,48].

### 5.2. Angiotensin-Converting Enzyme 2 (ACE2) Expression in Oral Tissues

**Figure 3:**
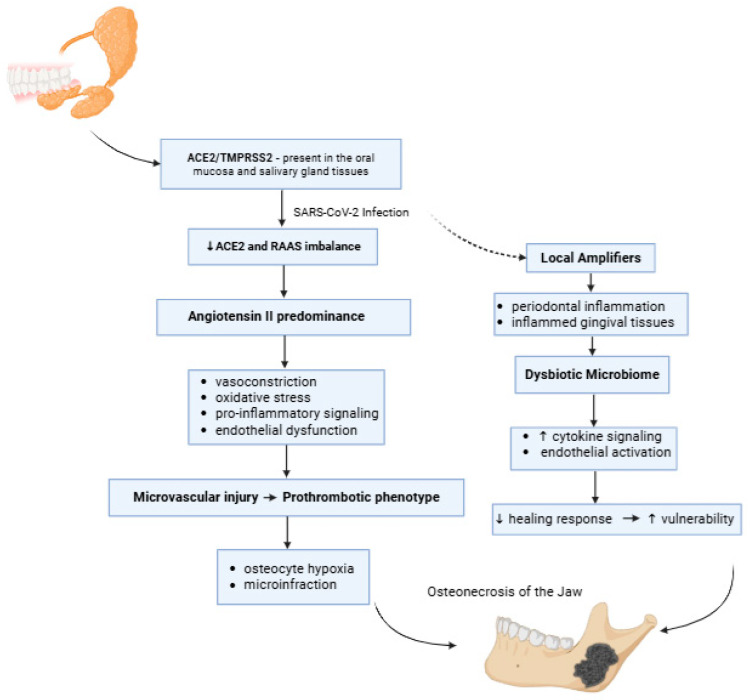
Representative scheme illustrating the proposed mechanism of post-COVID-19-related ONJ mediated by ACE2 expression in oral tissues. The scheme was made using Biorender.com (accessed on 18 January 2026; https://www.biorender.com/).

SARS-CoV-2 entry depends on host cell expression of entry factors. Multiple transcriptomic and protein-level studies demonstrate that ACE2 (often alongside TMPRSS2) is present in the oral mucosa and salivary gland tissues, supporting the oral cavity as a biologically plausible site of infection and local tissue injury (Figure 3). Single-cell and bulk expression analyses identified ACE2 enrichment in oral epithelial compartments (tongue epithelium and gingival/periodontal niches), suggesting that oral surfaces can function not only as viral exposure sites but also as permissive cellular targets. Salivary gland epithelial/ductal cells also express ACE2/TMPRSS2, and several studies propose salivary glands as potential sites of viral entry and replication, consistent with high viral loads detected in saliva during acute infection [49].

Mechanistically, oral ACE2 relevance extends beyond viral entry, because ACE2 is a key counter-regulatory enzyme of the renin–angiotensin–aldosterone system (RAAS). In physiological conditions, ACE2 degrades angiotensin II to angiotensin-(1–7), thereby favoring vasodilation, anti-inflammatory signaling, and endothelial stability. During SARS-CoV-2 infection, ACE2 can become functionally reduced (via internalization and/or shedding), shifting the RAAS balance toward angiotensin II predominance. This shift promotes vasoconstriction, oxidative stress, pro-inflammatory signaling, and endothelial dysfunction, which can amplify microvascular injury and a prothrombotic phenotype [50].

In the oral and maxillofacial context, the proposed sequence involves viral interaction with ACE2-expressing oral epithelium and/or salivary gland cells, the release of local inflammatory mediators and epithelial barrier perturbation, endothelial activation in adjacent microvasculature, and reduced vasoprotective RAAS signaling. Afterwards, impaired perfusion and reparative capacity in periosteal and intraosseous microcirculatory beds occur. Because alveolar bone is a high-turnover tissue with dense microvascular dependence, even modest, localized microcirculatory compromise may be sufficient to produce osteocyte hypoxia and microinfarction, lowering the threshold for clinically apparent osteonecrosis, especially when superimposed on systemic COVID-19 endotheliopathy/immunothrombosis or steroid exposure [51].

A further local amplifier is periodontal inflammation. Periodontal pockets and inflamed gingival tissues contain abundant immune cells and a dysbiotic microbiome. This environment can intensify cytokine signaling and endothelial activation and may increase vulnerability to viral-triggered injury. Accordingly, reviews discussing the COVID-19–periodontitis interface propose mechanisms involving direct infection susceptibility in periodontal tissues, local inflammatory priming, and worsened healing responses. Clinically, this framework is consistent with reports of oral ulcerations, worsened periodontal status, and delayed mucosal healing in subsets of COVID-19 patients, features that can facilitate mucosal breakdown over biologically compromised bone and promote secondary colonization [52,53].

Importantly, while ACE2 distribution supports plausibility, direct proof of persistent SARS-CoV-2 infection within the jawbone itself remains limited. Therefore, most authors frame ACE2-mediated effects as a mechanism for local susceptibility and vascular dysregulation rather than a confirmed direct cytopathic infection of osseous tissue [54].

### 5.3. Immune Dysregulation and Secondary Infection Susceptibility

**Figure 4:**
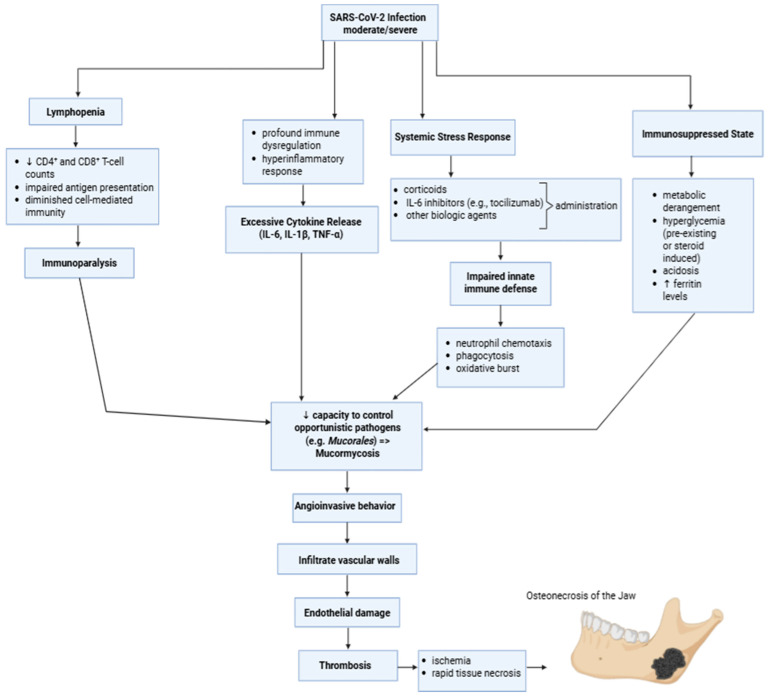
Representative schematic illustrating the proposed mechanism linking immune dysregulation following moderate-to-severe SARS-CoV-2 infection to the development of post-COVID-19 ONJ. The diagram summarizes the interplay between lymphopenia, cytokine-mediated hyperinflammation, systemic stress responses, and treatment-related immunosuppression, which collectively impair innate immune defenses and favor opportunistic infections such as mucormycosis. The angioinvasive behavior of these pathogens may lead to endothelial injury, thrombosis, tissue ischemia, and ultimately jaw osteonecrosis. Created with Biorender.com (Accessed on 18 January 2026; https://www.biorender.com/).

COVID-19, particularly in moderate to severe cases, is characterized by profound immune dysregulation (Figure 4) rather than simple immune activation or suppression. The acute phase of infection is often dominated by a hyperinflammatory response, marked by excessive cytokine release, including IL-6, IL-1β, and TNF-α, which can subsequently transition into a state of immune exhaustion or immunoparalysis [55,56]. Many hospitalized patients exhibit lymphopenia, with reduced CD4^+^ and CD8^+^ T-cell counts, impaired antigen presentation, and diminished cell-mediated immunity. This immune dysfunction may persist beyond clinical recovery, creating a window of transient but clinically significant immunosuppression [57,58].

In parallel, COVID-19 induces systemic stress responses and frequently necessitates immunomodulatory or immunosuppressive therapies (systemic corticosteroids, IL-6 inhibitors, and other biologic agents). While these treatments reduce mortality in severe disease, they further impair innate immune defenses, particularly neutrophil chemotaxis, phagocytosis, and oxidative burst activity. As a result, patients recovering from COVID-19 may be susceptible to opportunistic pathogens [59].

Immunosuppression is often amplified by metabolic conditions like hyperglycemia (either pre-existing or from steroid treatment), acidosis, and high serum ferritin levels, which can also lead to the growth of fungal pathogens. *Mucorales* species, for example, grow more intensely in environments rich in glucose and free iron. Therefore, COVID-19-associated mucormycosis is not a direct consequence of the virus’s action, but a secondary opportunistic infection, favored by the immune and metabolic dysregulation it induces [42,60,61,62]. *Mucorales* fungi exhibit angioinvasive behavior, penetrating vascular walls, causing endothelial injury, and facilitating thrombosis. After that, the vascular invasion leads to ischemia and rapid tissue death, especially in the sinonasal cavities and nearby maxillofacial structures like the maxilla and palate [42,63,64]. Similar, yet typically less aggressive mechanisms can be observed with other pathogens. Of these, *Aspergillus* species and various bacterial organisms, including *Staphylococcus aureus* and *Actinomyces*, can colonize and infect devitalized bone in the setting of impaired host defenses [5,65,66,67].

The jawbones tend to be vulnerable in this context owing to their proximity to the sinonasal tract, significant microbial exposure, and dependence on continuous microvascular perfusion for bone remodeling and repair. When immune surveillance is compromised, even minor breaches in the mucosa or already existing ischemic bone (e.g., from COVID-19-associated microthrombosis or steroid-related vascular injury) can serve as foci for secondary infection, leading to osteomyelitis and overt osteonecrosis [5,68].

Individuals who have survived COVID-19, especially older adults, people with diabetes, or those who received prolonged corticosteroid treatment, may carry lasting immunological and metabolic effects from the infection. This legacy lowers the risk of getting a second maxillofacial infection and helps explain why there have been so many cases of jaw osteonecrosis after COVID-19 in groups of people with similar immune, vascular, and metabolic risk factors.

### 5.4. Corticosteroid and Medication Effects

**Figure 5:**
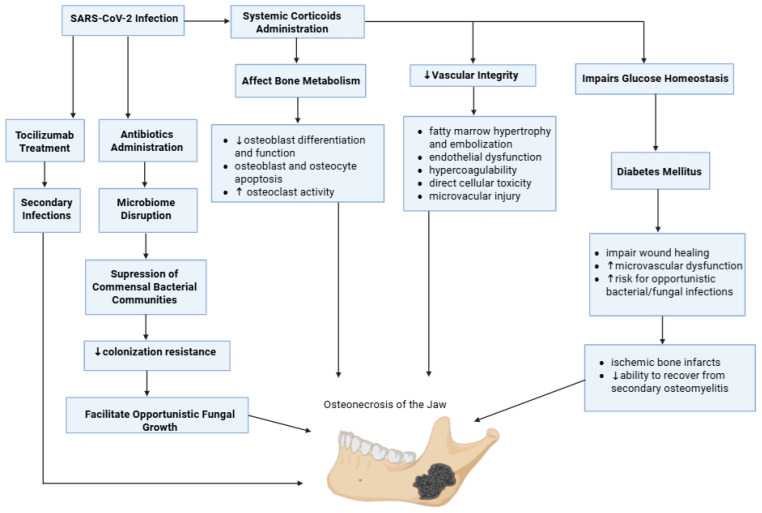
Representative schematic illustrating the proposed mechanisms linking SARS-CoV-2 infection and COVID-19-related treatments to the development of post-COVID ONJ. The diagram highlights the potential effects of systemic corticosteroid therapy and adjunct medications on bone metabolism, vascular integrity, glucose homeostasis, immune defense, and microbiome balance, which may collectively contribute to ischemic injury, impaired healing, and susceptibility to secondary infections. The scheme was made using Biorender.com (accessed on 18 January 2026; https://www.biorender.com/).

The widespread use of systemic corticosteroids in the treatment of COVID-19, most commonly dexamethasone at a dose of 6 mg daily for up to 10 days in hypoxic patients, has been identified as a major contributory factor in reported cases of PC-ONJ. While corticosteroids significantly reduce mortality in severe COVID-19, they exert well-known adverse effects on bone metabolism, vascular integrity, immune competence, and glucose homeostasis (Figure 5), which may collectively predispose susceptible individuals to jaw osteonecrosis [36,69].

Corticosteroids impair bone remodeling by inhibiting osteoblast differentiation and function, promoting osteoblast and osteocyte apoptosis, and altering osteoclast activity. These effects are central to glucocorticoid-induced skeletal fragility [69,70]. Prolonged or high-dose corticosteroid exposure is a recognized cause of avascular necrosis, classically affecting the femoral and humeral heads, through mechanisms that include fatty marrow hypertrophy and embolization, endothelial dysfunction, hypercoagulability, and direct cellular toxicity [69,71,72]. Although jaw involvement has been less frequently reported, it is plausible that even relatively short courses or moderate cumulative doses, particularly in patients with concurrent COVID-19-associated coagulopathy, endothelial dysfunction, or microvascular injury, could precipitate microvascular compromise or marrow embolic phenomena in the jaws, leading to delayed bone infarction weeks to months after exposure [71,72].

Besides their skeletal effects, corticosteroids exert potent metabolic consequences, most notably hyperglycemia. Dexamethasone can induce significant elevations in blood glucose levels and has been associated with new-onset diabetes mellitus in previously normoglycemic individuals [73,74]. During the pandemic, observational studies indicated that 50% of patients experienced steroid-induced hyperglycemia during treatment. It was found that patients undergoing extended treatment regimens or higher cumulative doses faced increased risk. The steroid-induced glycemia interacts with pre-existing diabetes, slowing wound healing, aggravating microvascular dysfunction, creating a metabolic environment favorable to opportunistic fungal infections. Hence, metabolic and vascular dysfunction together can make PC-ONJ more severe and contribute to its progression. This can occur by directly causing ischemic bone infarcts or by interfering with the organism’s ability to recover from secondary osteomyelitis [61,73,75,76].

Furthermore, corticosteroids exhibit immunosuppressive properties, reduce lymphocyte proliferation, and impair neutrophil chemotaxis, phagocytosis, and oxidative burst activity. Although these effects are advantageous in mitigating the cytokine release associated with COVID-19, they concurrently compromise the host’s defenses against bacterial and fungal pathogens, obscuring initial infection indicators. Consequently, in the post-COVID-19 context, this immune suppression can reactivate dormant odontogenic infections or enable opportunistic organisms to colonize ischemic or devitalized bone, promoting the advancement of osteomyelitis and osteonecrosis [59,77].

Other medications utilized in the initial stages of the pandemic might have inadvertently introduced supplementary, though indirect, risks via immune pathway suppression or microbiome disturbance. Tocilizumab, an IL-6 receptor antagonist administered in specific severe cases, has the potential to diminish inflammatory signaling, which is crucial for the antimicrobial host defense [78,79]. In clinical trials, IL-6 antagonists have been monitored for secondary infections. Meta-analytic trial data show secondary infection rates that are not uniformly increased across analyses. However, the risk of infection remains a recognized safety concern [80,81].

Broad-spectrum antibiotics, frequently administered empirically to hospitalized COVID-19 patients (especially early in the pandemic), may contribute to PC-ONJ via microbiome disruption. Suppression of commensal bacterial communities can reduce colonization resistance, facilitating opportunistic fungal overgrowth on mucosal surfaces (oral, sinonasal, and gastrointestinal). Reviews of COVID-associated fungal complications explicitly discuss how antibiotic exposure (often combined with steroids and diabetes) can promote dysbiosis and favor fungal proliferation/colonization, which is relevant to sinonasal fungal disease that can extend to the maxilla/palate and contribute to jaw necrosis in invasive cases [82,83,84]. Grillo et al. described patients who developed maxillary osteonecrosis several months after severe COVID-19 after exposure to high-dose corticosteroids, hydroxychloroquine, and prolonged broad-spectrum antibiotic therapy. The histopathological findings were consistent with osteomyelitis and devitalized bone rather than classical medication-related osteonecrosis patterns [85]. These observations support the hypothesis that secondary infection of ischemic or metabolically compromised bone, rather than direct drug toxicity alone, plays a central role in PC-ONJ. Hydroxychloroquine (HCQ), although no longer recommended for the treatment of COVID-19, possesses immunomodulatory properties that may have contributed to a post-COVID-19 susceptibility state, more specifically in early pandemic cohorts. HCQ accumulates within lysosomes, increases endosomal and lysosomal pH, interferes with antigen processing, and inhibits endosomal Toll-like receptor signaling (TLR7 and TLR9), resulting in attenuation of type I interferon production and downstream inflammatory cytokine responses. Even though existing clinical evidence does not strongly indicate a direct link between HCQ and osteonecrosis or a significantly elevated infection risk, its immunomodulatory properties could have functioned as a secondary co-factor during the post-acute recovery period. This is quite likely if HCQ diminished post-infectious immune surveillance and tissue repair capacity when combined with other contributing factors. These include systemic corticosteroid use, antibiotic-induced microbiome alterations, and the immune and vascular dysfunction associated with COVID-19. Consequently, within this complex, multifactorial context, HCQ is most appropriately considered a modifying factor, rather than a primary cause, of PC-ONJ [85,86].

### 5.5. Synergistic Comorbidities

**Figure 6:**
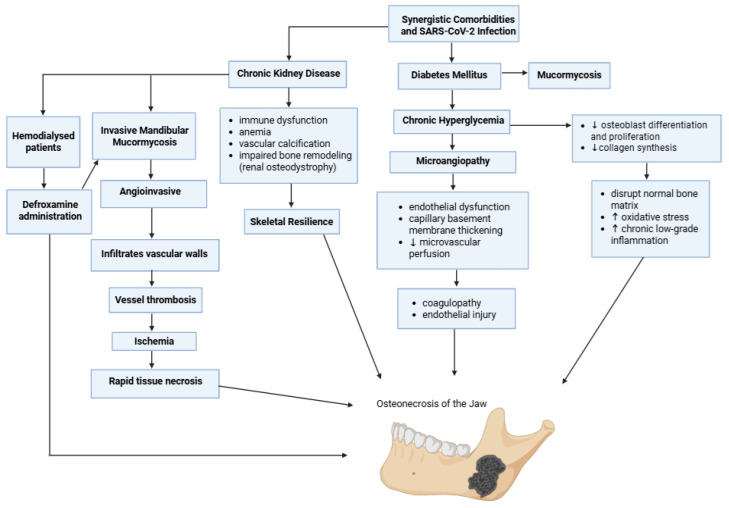
Representative scheme illustrating the proposed mechanism of post-COVID-19-related osteonecrosis of the jaw mediated by synergistic comorbidities. The scheme was made using Biorender.com (accessed on 18 January 2026; https://www.biorender.com/).

Pre-existing systemic comorbidities significantly exacerbate the pathophysiological processes that link COVID-19 to jaw osteonecrosis (Figure 6), being important modifiers of vascular integrity, immune function, and bone metabolism. These conditions do not act as isolated risk factors. Instead, they interact with COVID-19-induced endothelial damage, immunothrombosis, and treatment-related effects, thus diminishing the threshold for ischemic bone injury and impaired repair. Epidemiological evidence suggests that diabetes elevates the risk of mucormycosis by approximately threefold, especially in the context of COVID-19 and corticosteroid administration [61,62].

Chronic hyperglycemia leads to microangiopathy, characterized by endothelial dysfunction, thickening of the capillary basement membrane, and reduced microvascular perfusion. In long-standing diabetes, these changes affect blood flow in small vessels, including those supplying the alveolar bone and periosteum. When COVID-19-associated coagulopathy and endothelial damage overlap with diabetic microangiopathy, a synergistic impact is created, elevating the risk of localized ischemia and tissue infarction. In addition to its effects on blood vessels, hyperglycemia directly interferes with bone metabolism and healing processes. Experimental and clinical research shows that high glucose levels inhibit osteoblast proliferation and differentiation, reduce collagen synthesis, and disrupt normal bone matrix formation, while also promoting oxidative stress and low-grade chronic inflammation [7,76,87].

Besides diabetes, additional comorbid states may synergize with COVID-19 to predispose patients to jaw necrosis. Chronic kidney disease (CKD) is associated with immune dysfunction, anemia, vascular calcification, and impaired bone remodeling (renal osteodystrophy), all of which reduce skeletal resilience [88,89,90,91,92,93,94]. In post-COVID-19 settings, CKD has also been reported in association with destructive maxillofacial infections such as invasive mandibular mucormyscosis, which can directly produce jawbone necrosis through fungal invasion and vascular compromise [95]. In hemodialysis patients, exposure to deferoxamine is a recognized predisposing factor for mucormycosis, as the deferoxamine–iron complex serves as a xenosiderophore for *Mucorales*, enhancing fungal iron acquisition and growth. Importantly, mucormycosis is characteristically angioinvasive, infiltrating vascular walls and causing vessel thrombosis, which leads to ischemia and rapid tissue necrosis. When this process involves the rhino-maxillofacial region, the resulting perfusion failure can extend from sinonasal tissues into adjacent maxillary or mandibular bone, manifesting clinically as osteomyelitis and/or ONJ [42].

Ischemic risk may be exacerbated by other conditions linked to prothrombotic or antiangiogenic conditions. COVID-19-associated coagulopathy, which is marked by endothelial dysfunction, thromboinflammation (immunothrombosis), and micro-/macrovascular thrombosis, can induce tissue hypoperfusion and infarction in vulnerable vascular regions. In individuals with a history of thrombophilias, such as antiphospholipid syndrome, the genetic predisposition to microvascular thrombosis and endothelial activation provides a mechanistic explanation for an increased risk when combined with COVID-19-related immunothrombosis. Nevertheless, the extent of this interaction in post-COVID-19 jaw necrosis specifically is still unclear and is best considered a possible susceptibility modifier rather than a strong established association [2]. Similarly, antiangiogenic cancer therapies (e.g., VEGF/VEGFR pathway inhibitors) can impair microvascular repair and mucosal–osseous healing and have been implicated as co-medications in MRONJ, particularly in combination with antiresorptive agents. In the setting of COVID-19 endotheliopathy, such therapies could plausibly contribute to a “dual hit” on perfusion and regeneration, although direct PC-ONJ data remain limited [96].

Taken together, these observations support the concept that COVID-19 functions as a force multiplier rather than a solitary etiologic agent. In individuals with underlying metabolic, vascular, or immunologic vulnerability, SARS-CoV-2 infection and its systemic sequelae, along with the therapies used to treat it, can convert a background predisposition into active pathology. This framework helps explain why PC-ONJ disproportionately affects patients with overlapping comorbidities and underscores the importance of heightened vigilance and risk stratification in COVID-19 survivors.

## 6. Clinical Presentation and Spectrum of Post-COVID-19 Jaw Osteonecrosis

### 6.1. General Clinical Framework and Common Features

The COVID-19 pandemic has brought to the attention of the medical community a series of previously rare or insufficiently characterized oro-maxillofacial manifestations, including osteonecrotic involvement of the jaws following infection with severe acute SARS-CoV-2. Analysis of the literature shows that these lesions cannot be classified as a single clinical entity but represent a spectrum of clinical and imaging presentations with overlapping phenotypes but different etiological mechanisms [5,84,97].

In this context, the term PC-ONJ is used as a clinical descriptor, not as a definitive etiological diagnosis, and reflects a convergence of ischemic, inflammatory, and infectious processes, favored by the post-viral context and COVID-19-associated treatments [98,99].

Regardless of etiology, recurrent clinical manifestations reported transversally in the literature include persistent orofacial pain, local swelling, sudden or progressive tooth mobility, exposed or probable bone, oroantral or oronasal fistulas, and oral ulcerations with delayed healing [5,6,7,100].

A distinctive clinical feature is that symptoms may precede bone exposure by weeks or months, unlike classic presentations of MRONJ, where exposed bone is often the dominant initial sign [5,98].

### 6.2. Maxilla Versus Mandible: Patterns of Involvement and Clinical Implications

A consistent finding in the literature is the clear predominance of maxillary involvement, reported in over 90% of cases, while the mandible is involved much less frequently (≈8–9%) [5,6,99].

This pattern is atypical compared to classic odontogenic osteomyelitis, where the mandible is the dominant location, and supports the existence of a distinct post-COVID-19 pattern, possibly influenced by the proximity of the maxilla to the paranasal sinuses, the particularities of local vascularization, and the increased susceptibility to ischemia and sinonasal extension [97,98].

Involvement of the paranasal sinuses is reported in approximately 60% of cases and is an indirect marker of extension and severity, especially when it occurs early or is associated with ENT (ear, nose, and throat) symptoms [5,6]. The imaging changes described include sinus mucoperiosteal thickening, sinus wall erosions, and extension to adjacent structures [6,7].

Although rare, mandibular involvement has been documented both in isolation and in association with maxillary involvement, indicating that, at the individual level, presentations may be mixed, complicating the differential diagnosis [6,85].

### 6.3. Timeline of Onset: Acute Onset Versus Late Presentations

The interval between SARS-CoV-2 infection and the onset of oro-maxillo-facial manifestations is highly variable. The reported mean is approximately 100 days, with a wide range from onset concurrent with active infection to more than 12–21 months post-COVID-19 [5,6].

Early-onset forms are more commonly associated with CAM and present with rapid, aggressive progression with sinonasal, orbital, or intracranial extension [66,100,101].

In contrast, late presentations are characterized by nonspecific dentoalveolar symptoms, which favor initial interpretation as common dental pathology or chronic osteomyelitis [6,85,102].

Importantly, the latency of onset does not imply a milder form but reflects a subacute or insidious evolution of the pathological process [5].

### 6.4. Late Presentations and Overlooked Cases

A recurring element in published series is late diagnosis. The main reasons include initial absence of bone exposure, attribution of pain and dental mobility to common dental causes, and nonspecific imaging in the early stages [6,67,102].

There are reports of cases treated for a long time as bacterial osteomyelitis, with repeated antibiotic therapy, before the appearance of bone sequestration or oro-sinus communications [6,85]. In other situations, tooth extractions acted as triggering events, with necrosis occurring months after the acute episode of COVID-19 [6,7].

These delayed presentations underscore the need to maintain clinical suspicion in patients with a history of COVID-19 who develop persistent pain, unexplained tooth mobility, or unusual mucosal lesions, even in the absence of “classic” signs of osteonecrosis.

### 6.5. Integrating the Clinical Spectrum: Implications for Practice

The available data support the existence of a continuous clinical spectrum (Table 2), with presentations ranging from slowly progressive, predominantly dentoalveolar forms to invasive presentations with sinonasal, orbital, or cerebral extension [3,5,100].

For the clinician, the key message is that a history of SARS-CoV-2 infection should be viewed as a contextual risk factor, requiring increased vigilance and staged evaluation, rather than as a definitive diagnosis. The correct approach involves integrating symptomatology, anatomical distribution, and chronology, with etiological confirmation through histopathological and microbiological investigations when the evolution is atypical or aggressive [6,99].

Furthermore, Table 3 summarizes the main demographic characteristics, comorbidities, and COVID-19-related clinical factors reported in patients diagnosed with post-COVID-19 jaw osteonecrosis.

## 7. Diagnostic Challenges and Dilemmas

### 7.1. Clinical Context: Why the Diagnosis of Post-COVID-19 Oro-Maxillofacial Lesions Remains Difficult

Following the COVID-19 pandemic, it has become increasingly evident that oro-maxillofacial lesions occurring after SARS-CoV-2 infection may present with overlapping clinical and radiological phenotypes, despite different underlying etiopathogenic mechanisms [5,6,32,100].

In this context, the term PC-ONJ has been used to describe a diagnostic “borderline” area, in which the etiological diagnosis cannot be established on the basis of a single clinical or imaging feature, but rather requires a step-wise integrative approach [5,7,104].

Dedicated case series and reviews consistently report that symptoms such as orofacial pain, swelling, sudden tooth mobility, exposed or probing bone, fistula formation, and sinonasal manifestations may occur across different pathological entities, including chronic osteomyelitis, MRONJ, ORN, and invasive fungal disease, explaining why simplistic pattern recognition may lead to classification errors [6,32,100].

Moreover, several authors underscore that, in ambiguous cases, etiological decision-making should rely on the combined triad of imaging for extension assessment, biopsy for etiological clarification, and targeted microbiological investigations for confirmation and therapeutic guidance, in order to avoid misclassification and inappropriate management [11,103,105,106,107].

### 7.2. Imaging Findings: Essential Role in Assessing Extension, Limited Value in Establishing Etiology

The literature and current clinical practice consistently support that imaging investigations are indispensable for assessing and delimiting the extent of oro-maxillofacial lesions, but in most cases, they do not allow the exact etiology of the pathological process to be established. The authors note that imaging changes such as osteolysis, the presence of bone sequestration, or cortical destruction can be found in a variety of different entities, including chronic osteomyelitis, MRONJ, ORN, CAM, or post-viral bone necrosis phenotypes, without being pathognomonic for any of them.

In current practice, orthopantomography (OPG) is often the first imaging investigation performed, as it is useful for identifying areas of osteolysis and bone fragments suggestive of sequestration. However, the authors point out that OPG has significant limitations in the early stages of the disease and in situations where the damage is predominantly medullary, cases in which changes may be absent or discreetly expressed.

For this reason, several studies recommend rapid escalation to advanced imaging investigations, such as cone-beam computed tomography (CBCT) or computed tomography (CT), especially when clinical symptoms are disproportionate to the initial radiological examination or when associated sinonasal signs are present. These methods allow for a more accurate three-dimensional assessment of bone extension and its relationship to adjacent structures, but the authors emphasize that neither CBCT nor CT can definitively differentiate between ischemic, bacterial, or fungal etiology of the lesions.

In the case series reported by Al-Mahalawy et al., CT was mainly used to delineate the anatomical extent of bone lesions and to assess their relationship with the maxillary sinus, respectively to assess the degree of sinus occupancy or permeability, without being considered a decisive method for establishing the etiology of the pathological process.

In the same context, the authors note from a methodological point of view that CT plays an essential role in the anatomical assessment of lesions, allowing the assessment of bone destruction and maxillary sinus involvement, but does not allow etiological differentiation between an ischemic, bacterial, or fungal process, nor does it exclude malignancy [7].

For the evaluation of soft tissues, magnetic resonance imaging (MRI) is described as useful in assessing the extent of lesions and the severity of loco-regional involvement, particularly at the sinus, orbital, or adjacent soft tissue levels. However, the authors insist that MRI cannot replace biopsy when the etiological suspicion remains open, as it does not provide histopathological confirmation [7].

In this regard, Kang et al. describe a representative case in which OPG was initially non-suggestive, despite intense pain and marked dental mobility, highlighting an important diagnostic pitfall. The authors point out that, in such situations, delaying advanced imaging investigations can lead to underdiagnosis or late establishment of the correct diagnosis [67].

### 7.3. Biopsy and Microbiology

In studies analyzing non-fungal phenotypes of post-COVID-19 osteonecrosis-COVID-19, Al-Mahalawy at al. propose a differentiated diagnostic pathway designed to separate secondary bacterial colonization from invasive fungal infection in order to avoid misclassifying aseptic necrosis as mucormycosis (or, conversely, underdiagnosing an invasive infection) [7].

In this regard, the authors describe separate microbiological sampling, with routine bacterial cultures and dedicated fungal cultures (Sabouraud medium, with prolonged incubation of at least 7 days), illustrating the need for an etiologically oriented microbiological approach rather than a single, non-specific sampling [7].

In line with this approach, international guidelines for the management of mucormycosis associated with COVID-19 underscore that a definitive diagnosis cannot be established based on imaging, which plays an exclusive role in assessing the extent of the lesions, and that etiological confirmation is based on histopathological demonstration of fungal hyphae and vascular angioinvasion.

Rudramurthy et al. describe that direct microscopic examination of tissue material or exudate using potassium hydroxide (KOH), with or without calcofluor staining, is a rapid and useful method for identifying hyphae characteristic of *Mucorales* infections, namely broad, pauciseptate, or aseptate hyphae with right-angled branching [105].

However, the same authors note that etiological confirmation cannot be based solely on microscopic examination, as histopathology provides essential additional information, frequently demonstrating vascular thrombosis, ischemic necrosis, and hemorrhagic infarcts, changes that cannot be reliably differentiated based solely on clinical examination or imaging investigations, including CT [105].

Regarding microbiological diagnosis, Rudramurthy points out that samples intended for culture must be handled with care, as *Mucorales* hyphae are fragile and can be destroyed during processing. In cases where the results are inconclusive, the authors recommend the use of molecular techniques (PCR) from fresh tissue as a complementary method of diagnostic clarification [105].

The diagnostic value of integrated tissue assessment is illustrated by the report by Romano et al., in which microbiological examination of the exudate revealed bacterial cultures of *Aspergillus niger* and *Staphylococcus epidermidis*, while serum fungal markers (β-D-glucan and galactomannan) remained within normal limits. This discrepancy highlights the risk of incomplete interpretation when using a single type of diagnostic test [104].

In the same case, histopathological examination with Grocott staining revealed the presence of aseptate hyphae compatible with mucormycosis, and the authors also describe septate structures with dichotomous branching, suggesting the possibility of a fungal co-infection. These observations support the role of biopsy as a central tool for etiological clarification, especially in cases with an ambiguous clinical and microbiological presentation [104].

### 7.4. Differential Diagnosis: Clinical and Imaging Overlaps

Al-Mahalawy et al. highlight that in the post-COVID-19 context, atypical forms of maxillary or mandibular necrosis may occur that clinically and imaging-wise overlap with multiple already known entities, making it impossible to differentiate etiologically based on clinical examination alone. The authors show that the same clinical picture can mimic odontogenic osteomyelitis, complicated maxillary sinusitis, MRONJ-like phenotypes, ORN, or COVID-19-associated invasive fungal infection (CAM/ROCM), requiring a systematic approach to differential diagnosis [7,104].

Romano et al. illustrate this diagnostic challenge by describing cases in which maxillary osteonecrosis, most likely driven by ischemic mechanisms, coexists with or is difficult to differentiate from invasive fungal osteomyelitis within the same clinical presentation. In such situations, the authors emphasize that the diagnosis must be established affirmatively through targeted and staged investigations rather than assumptions based on clinical evolution or response to empirical treatment. They further describe the frequent overlap between severe rhinosinusitis and maxillary or palatal bone extension, often accompanied by oroantral or oronasal fistulas. In these cases, the dominant symptoms may be ENT-related, which can delay dento-maxillary evaluation and lead to underestimation of bone involvement. The authors also highlights the risk that relevant etiological exposures, such as antiresorptive or antiangiogenic therapies or a history of cervicofacial radiotherapy, may be overlooked during medical history taking, potentially resulting in incorrect classification as MRONJ or ORN with direct implications for therapeutic management. Finally, the author stresses the essential role of biopsy not only in clarifying the infectious etiology but also in excluding malignancy, particularly in cases characterized by extensive bone destruction, persistent fistulas, and exuberant granulation tissue, especially when imaging findings suggest aggressive disease behavior [106].

In an analysis focused on entities that can mimic post-COVID-19 osteonecrosis, several authors warn of diagnostic errors frequently encountered in practice: interpreting necrosis as simple bacterial osteomyelitis, with the institution of prolonged and ineffective antibiotic therapy; confusion of osteolytic lesions with bone metastases; and underestimation of the extent of soft tissue involvement when the assessment is based solely on CT, without supplementation by MRI.

### 7.5. ENT Perspectives Versus Perspectives from the Field of Dentistry/OMFS: Interdisciplinary Dilemmas and the Need for Coordinated Assessment

The literature and recent guidelines highlight that patients with COVID-19-associated maxillary osteonecrosis may initially present to different services, depending on their dominant symptoms. Thus, some patients arrive at ENT services for sinonasal manifestations, such as unilateral rhinorrhea, nasal congestion, cacosmia, or facial pain, while others are initially evaluated by oral and maxillofacial surgery (OMFS) specialists for dental pain, sudden tooth mobility, or bone exposure. This fragmentation of the initial pathway may delay recognition of the pathological process as a single entity.

Rudramurthy et al. indicate in the CAM management guidelines that accurate diagnosis and effective management require a multidisciplinary approach. The authors point out that the integration of ENT clinical data with oro-maxillary signs and extensive imaging investigations is essential for accurately delineating the extent of the disease and avoiding underestimation of bone involvement [105].

In this context, Omranzadeh and other authors describe imaging as a central element of communication between specialties, as CT investigations can demonstrate the anatomical continuity between paranasal sinus involvement and maxillary bone destruction. However, the authors insist that image interpretation must be performed in strict correlation with the integrated clinical picture, as radiology alone, in the absence of interdisciplinary evaluation, cannot establish the aetiology of the process [103].

Datarkar et al. highlight the risk that patients may initially be diagnosed and treated for severe sinusitis, while maxillary necrosis is recognized late. In this regard, the authors recommend early dental or OMFS evaluation in situations where atypical signs appear, such as sudden tooth mobility, palatal defects, or bone exposure, even if the initial symptoms are predominantly ENT-related [100].

Romano et al. provide an illustrative example of this interdisciplinary discrepancy, describing a case in which the initial presentation was dominated by ENT symptoms such as unilateral rhinorrhea, cacosmia, frontal headache, and persistent cough, while the oro-maxillofacial examination revealed oro-nasal communication, oro-antral fistula with purulent exudate, and extensive necrotic bone exposure. This case highlights the need for systematic correlation of clinical findings from both fields [104].

Following the same logic, the authors show that effective management often involves a combination of interventions specific to each specialty, such as sequestrectomy or debridement performed by the OMFS surgeon, associated with ENT endoscopic procedures, such as functional endoscopic sinus surgery (FESS). This integrated approach demonstrates that a rigid separation of responsibilities between specialties can delay control of the pathological focus and negatively influence the prognosis [104].

### 7.6. Diagnostic Algorithm and Warning Signs: Preventing Misclassification

Given the variety of clinical manifestations and frequent overlaps with chronic osteomyelitis, MRONJ, ORN, or malignant pathology, the literature recommends adopting a stepwise diagnostic algorithm. This approach should be based on the systematic correlation of clinical, imaging, and histopathological data, avoiding the premature attribution of an etiology based on a single type of investigation.

Several authors warn that the lack of a standardized diagnostic framework favors two major types of errors: underdiagnosis of invasive fungal infections with severe potential progression and overdiagnosis of CAM in cases of non-fungal bone necrosis occurring post-COVID. This confusion has direct implications for treatment choice and patient prognosis.

In this context, Sourani and Datarkar identify a series of clinical warning signs that require reassessment and a progressive diagnostic approach. These include persistent or disproportionate facial pain compared to the initial clinical findings, progressive swelling, sudden tooth mobility, persistent bone exposure, the presence of oroantral or oronasal fistulas, and foul-smelling secretions. The appearance of these elements suggests an active pathological process and warrants further in-depth investigation [11,100].

Khan at al. bring attention to the fact that the absence of a clinical response to empirical antibiotic therapy should be interpreted as a diagnostic red flag. The authors insist that this situation does not justify prolonging conservative treatment without etiological confirmation but requires reconsideration of the initial diagnosis and completion of the evaluation using invasive methods when necessary [6].

Within the same algorithm, the authors highlight the role of advanced imaging, such as CT and MRI, in assessing the extent and severity of lesions. However, the literature underscores that these methods cannot establish the etiology of the process in ambiguous cases. Etiological confirmation remains dependent on histopathological examination and targeted microbiological investigations, which allow differentiation between ischemic necrosis, bacterial infection, and invasive fungal infection [6].

To reduce diagnostic ambiguity and avoid misclassification, a structured diagnostic workflow for suspected PC-ONJ is proposed and summarized in Table 4.

In Table 5, a structured diagnostic algorithm for PC-ONJ and CAM-ROCM is presented, outlining the essential diagnostic stages, frequent diagnostic errors, and the practical role of each step in guiding clinical decision-making.

### 7.7. Operational Diagnostic Considerations for Distinguishing PC-ONJ from Other Jaw Necrosis Entities

Although universally validated diagnostic criteria for PC-ONJ are not yet available, the current literature allows the formulation of an operational rule-out framework that may guide clinical reasoning in suspected cases. In practice, PC-ONJ should only be considered after systematic exclusion of major overlapping entities known to cause osteonecrosis of the jaw. The diagnostic process therefore relies on a structured exclusion of alternative etiologies, supported by clinical history, imaging, microbiology, and histopathology when indicated.

The following considerations may serve as a practical diagnostic framework:

**1.**  **Exclusion of MRONJ**

The absence of exposure to antiresorptive or antiangiogenic agents should be carefully documented in the patient’s medical history. This includes bisphosphonates, denosumab, and antiangiogenic therapies used in oncology or other chronic conditions. Because MRONJ remains the most prevalent non-radiation-related cause of jaw osteonecrosis, failure to identify such exposures may lead to incorrect attribution of the lesion to a post-COVID-19 mechanism. A thorough medication history is therefore essential before considering a diagnosis of PC-ONJ.

**2.**  **Exclusion of ORN**

Prior cervicofacial radiotherapy must be ruled out through careful review of the patient’s oncologic history. Osteoradionecrosis typically develops within irradiated bone territories and is strongly associated with radiation-induced vascular compromise and impaired tissue repair. In the absence of documented radiotherapy affecting the maxillofacial region, ORN becomes unlikely, allowing clinicians to consider alternative etiologies.

**3.**  **Exclusion of invasive fungal disease, particularly CAM**

Given the well-documented association between COVID-19, immunosuppression, corticosteroid therapy, and invasive fungal infections, the possibility of fungal osteomyelitis must be carefully evaluated. When clinical or radiologic features suggest aggressive disease, such as rapid progression, sinonasal involvement, or orbital extension—tissue biopsy with histopathological examination becomes essential. Demonstration of fungal hyphae with angioinvasion remains the diagnostic gold standard. Targeted microbiological studies and fungal cultures may provide complementary information, although negative cultures do not exclude invasive disease. Only after invasive fungal infection has been reasonably excluded should a non-fungal mechanism such as PC-ONJ be considered.

**4.**  **Exclusion of primary chronic bacterial osteomyelitis**

Chronic bacterial osteomyelitis represents another important differential diagnosis and may mimic the radiologic and clinical features of osteonecrosis. Distinguishing features may include purulent drainage, clear odontogenic infection sources, and microbiological evidence of bacterial pathogens. Correlation between clinical presentation, microbiological findings, and therapeutic response to antibiotics is, therefore, necessary. In cases where infection appears secondary to necrotic bone rather than the primary driver of disease, the diagnosis may shift toward a necrotic-ischemic process rather than classical osteomyelitis.

**5.**  **Exclusion of malignancy in destructive or atypical lesions**

In situations characterized by extensive bone destruction, persistent fistulas, atypical granulation tissue, or radiologic features suggestive of aggressive disease, malignancy must be excluded. Biopsy is therefore indicated to rule out primary bone tumors, metastatic disease, or malignancies originating in adjacent structures such as the maxillary sinus. This step is particularly important in cases where imaging demonstrates infiltrative or rapidly progressive lesions.

**6.**  **Identification of supportive, yet non-pathognomonic features suggestive of PC-ONJ**

Once the major alternative etiologies have been excluded, several clinical and contextual elements may support the consideration of PC-ONJ. These include a temporally plausible history of SARS-CoV-2 infection occurring weeks to months before symptom onset, a predominance of maxillary involvement reported in several case series, delayed presentation after the acute viral illness, and bone changes compatible with ischemic necrosis and sequestration. Additional systemic factors frequently described in affected patients include diabetes mellitus, prolonged corticosteroid exposure, endothelial dysfunction, and microvascular alterations associated with severe COVID-19.

Within this framework, PC-ONJ should therefore be regarded as a diagnosis of exclusion supported by a compatible clinical context rather than a condition defined by pathognomonic criteria. This approach reflects the current state of knowledge and acknowledges the ongoing debate regarding the exact pathophysiological mechanisms underlying post-COVID-19 jaw osteonecrosis.

## 8. Management and Treatment Strategies in Post-COVID-19-Related Osteonecrosis of the Jaw and COVID-Associated Mucormycosis

### 8.1. Medical Management

#### 8.1.1. Basic Principle: Etiologically Guided Treatment (CAM vs. PC-ONJ)

The medical management of maxillary osteonecrosis occurring in the context of COVID-19 must be adapted to the dominant etiology of the lesion, clearly differentiating between fungal (CAM) and non-fungal (PC-ONJ) forms, as this distinction has major implications for further therapeutic approaches: administration of antifungal/antibiotic therapy and the intensity of systemic monitoring [7,105,106].

#### 8.1.2. Antifungal Therapy in CAM

The authors of the ECMM-MSG ERC (European Confederation of Medical Mycology—Mycoses Study Group Education and Research Consortium) guide emphasize that mucormycosis is a medical-surgical emergency, in which early initiation of antifungal therapy directly influences survival, with overall mortality reported between 40 and 80%, depending on the extent of the disease and the patient’s immune status [106].

Liposomal amphotericin B (L-AmB) is recognized as first-line therapy, with recommended doses of 5–10 mg/kg/day, higher doses being reserved for cases with brain involvement or rapid progression [106,107]. Muthu et al. also report the use of L-AmB as initial treatment in CAM, emphasizing the need for strict monitoring of renal function and electrolytes [107].

Yasmin et al. show that delaying the initiation of amphotericin B by ≥6 days after diagnosis was associated with an increase in mortality from 49% to 83% at 12 weeks, highlighting the critical role of timing and therapeutic decision-making in CAM [110].

Isavuconazole and posaconazole are described as rescue or step-down therapy options, especially in patients with renal toxicity or contraindications to amphotericin B, their efficacy being reported as comparable to the therapy of choice in the available data [106,110].

#### 8.1.3. Medical Management in Non-Fungal PC-ONJ

In contrast, in forms of post-COVID-19 osteonecrosis without fungal confirmation, the authors point out the need to avoid empirical initiation of antifungal therapy in the absence of histopathological or microbiological evidence, both for therapeutic accuracy and for the principles of rational use of antimicrobials [7,16].

The case series reported by Al-Mahalawy et al. (12 patients) indicates that management based on perioperative antibiotic therapy and surgical debridement, without antifungals, may be effective in the absence of confirmed mucormycosis, supporting a model of antimicrobial stewardship applicable to PC-ONJ [7].

Broad-spectrum antibiotics are recommended only in the presence of signs of bacterial superinfection, and antiseptic rinses with 0.12–0.2% chlorhexidine remain a standard component of conservative treatment [16].

### 8.2. Surgical Management

#### 8.2.1. The Role of Surgery and the Principle of Individualization

Surgical intervention is an essential pillar of management, but its extent and timing must be individualized according to etiology, extent of necrosis, response to medical treatment, and the patient’s general status [24,106,111]. The authors consistently draw attention to the fact that the surgical approach in PC-ONJ/CAM must be adapted according to local severity and the risk of loco-regional progression, especially in the context of associated fungal infection or lack of response to conservative treatment [11,100].

#### 8.2.2. Definition and Role of Sequestrectomy

Sequestrectomy is a surgical procedure for the selective removal of necrotic bone fragments (sequestra) delimited by vital bone, with the aim of reducing the local infectious load, removing devitalized tissue, and facilitating healing by exposing a viable bone bed. According to the authors, sequestrectomy is a conservative surgical procedure, indicated when the residual bone has bleeding/viability potential and when the anatomical extension allows structural preservation [11,100].

#### 8.2.3. Conservative Surgery: Debridement and Sequestrectomy

In cases with necrosis limited to the dental alveolus or restricted portions of the jaw, surgical debridement and sequestrectomy are the first-line options, especially in non-fungal PC-ONJ or in forms without sinus/orbital extension [6,11]. In the systematic analysis by Sourani et al., all 12 patients included underwent surgery (predominantly debridement and sequestrectomy) associated with antibiotic therapy, with a clinical resolution rate of 87% in patients with available follow-up [11].

In the same dataset, maxillary involvement is reported in 83.3% of patients, and surgical treatment by debridement (with or without associated extractions) was used systematically, supporting the effectiveness of this approach in non-extensive forms [11]. In other series, in 113 patients with maxillary osteomyelitis associated with post-COVID-19 mucormycosis, sequestrectomy was performed in 14% of cases, being reserved for patients with localized lesions without major structural involvement [100].

#### 8.2.4. Radical Surgery: Extensive Resections

In advanced forms, characterized by extensive bone destruction and sinus/palatal involvement or oro-nasal communications, the authors describe the need for aggressive surgical approaches. In CAM, wide resection is considered essential for disease control due to the angioinvasive nature of fungi of the order *Mucorales*, which favor rapid progression despite drug therapy if debridement is insufficient [100,112].

Mohebbi et al. describe a case of late maxillary bone sequestration that required resection of the hard palate, pterygoid plates, and adjacent teeth, followed by prosthetic reconstruction, due to persistent symptoms and histopathological confirmation of mucormycosis [112]. In another case study, the distribution of interventions reflects the severity of the disease: bilateral maxillectomy 10%, hemimaxillectomy 5%, partial maxillectomy 5%, isolated palatal resections 2%, illustrating the need for surgical escalation depending on anatomical extension [100].

#### 8.2.5. Criteria for Timing and Extent; Postoperative Complications

The timing of intervention is presented as a major prognostic factor. The authors emphasize that early intervention, after metabolic stabilization and initiation of antifungal therapy, is associated with more favorable outcomes and lower mortality. In CAM, delayed intervention may allow progression to the orbit or brain; in the series by Datarkar et al., the reported mortality was 1.76%, with deaths limited to patients with brain involvement, which supports the importance of adequate timing [100,112].

#### 8.2.6. Postoperative Complications

Regarding complications, several cases report postoperative events in 7/12 patients, including oroantral communications and persistent inflammation, which requires careful monitoring after local interventions [11]. Also, there are complications such as dehiscence 11%, pain/edema 7%, and suppuration 6% reported, highlighting the functional cost of interventions and the need for multidisciplinary follow-up [100].

### 8.3. Adjunctive Therapies

Current studies highlight the role of adjuvant therapies in optimizing healing, reducing inflammation, and lowering the risk of recurrence, without substituting basic medical and surgical treatment.

#### 8.3.1. Hyperbaric Oxygen Therapy (HBOT)

HBOT is described as a therapy capable of improving tissue oxygenation, stimulating angiogenesis and osteoblastic activity, being extrapolated from the management of ORN and MRONJ to contexts such as PC-ONJ/CAM, especially in patients with hypoxia and delayed healing [16,111]. Also, HBOT may be considered in advanced stages as an adjunct to debridement to facilitate re-epithelialization and reduce the risk of secondary infection. Specific data on PC-ONJ remain limited, but the authors suggest selective use based on common mechanisms (ischemia, microvascular thrombosis, chronic inflammation) [16,112].

HBOT is typically administered at 2.0–2.5 ATA for approximately 90 min per session, usually delivered in 20–30 sessions, with additional postoperative sessions when surgical treatment is performed, according to protocols originally described for osteoradionecrosis and later applied to other forms of jaw osteonecrosis [113,114].

#### 8.3.2. Local Antiseptics and Controlled Oral Hygiene

Oral rinses with 0.12–0.2% chlorhexidine are consistently reported as a basic adjuvant measure in conservative and postoperative management, reducing bacterial load and limiting superinfection in areas with exposed bone [11,16]. Datarkar et al. mention the use of oral antiseptics in multimodal protocols alongside antibiotics/antifungals and surgery as part of standardised local care [100].

#### 8.3.3. Ozone Therapy

Ozone therapy is discussed in particular in MRONJ, with potential extrapolation to PC-ONJ, through antimicrobial, anti-inflammatory, and biostimulant effects. Rincon et al. describe the use of ozone (gas/ozonated water) associated with symptom relief and improved clinical status in MRONJ, suggesting adjuvant utility in non-fungal or residual post-COVID-19 osteonecrosis in carefully selected contexts [115].

#### 8.3.4. Platelet-Rich Plasma (PRP)

PRP is presented as an adjuvant biological therapy capable of supporting regeneration through the release of growth factors. Longo et al. describe the use of PRP in bisphosphonate-associated osteonecrosis, highlighting effects on angiogenesis and osteogenesis. Extrapolating to PC-ONJ, the authors suggest the potential of PRP in post-sequestrectomy or post-resection defects, especially in patients with compromised vascular status post-COVID-19 [116].

#### 8.3.5. Low-Level Laser Therapy (LLLT)/Photobiomodulation

Hanna et al. analyze laser photobiomodulation, noting anti-inflammatory, analgesic, and biostimulating effects, with applicability in MRONJ and other chronic lesions. The authors report the possibility of reducing postoperative pain and accelerating mucosal healing, although they underline the need for standardization of protocols and dedicated studies for targeted application [117].

#### 8.3.6. Multimodal Integration

Clinical syntheses converge on the idea that adjuvant therapies are most effective in multimodal, patient-tailored protocols, in which local therapy is combined with systemic treatment and surgery, aiming to reduce recurrence and improve healing [11,100].

### 8.4. Multidisciplinary Care

The complexity of PC-ONJ/CAM requires a structured multidisciplinary approach, and the lack of coordination between specialties is described as a major cause of delay in correct diagnosis and progression to severe forms [5,6].

#### 8.4.1. Oral and Maxillofacial Surgeon

The oral and maxillofacial surgeon is responsible for assessing the extent of necrosis, surgical indication, and coordinating local stages. Also, it is important to differentiate between chronic osteomyelitis, MRONJ, and CAM for the correct choice of strategy. The timing and extent of debridement/resection should be correlated with systemic status and response to antifungal/antibiotic therapy [100].

#### 8.4.2. ENT Specialist

The involvement of the ENT specialist is indispensable in sinus/nasal/orbital extensions. The involvement of the sinus may be underdiagnosed if the evaluation remains strictly dento-alveolar, and the role of the ENT includes imaging interpretation, endoscopic biopsies, and sinonasal debridement to reduce the fungal load [101,103].

#### 8.4.3. Infectious Disease Specialist

The guidelines point out the role of the infectious disease specialist in choosing the antifungal agent, adjusting doses, and monitoring toxicity, with a direct impact on survival in CAM [105,106]. In non-fungal PC-ONJ, their contribution extends to optimizing antibiotic therapy and differentiating bacterial superinfection from “sterile” necrosis [7].

#### 8.4.4. The Endocrinologist and Internist

Metabolic imbalances, especially uncontrolled diabetes mellitus, are associated with unfavorable outcomes. Muthu et al. and Yasmin et al. describe an increased prevalence of hyperglycemia/diabetes in CAM patients and demonstrate that lack of metabolic control compromises the response to antifungal therapy and surgical outcomes, which is why the endocrinologist is essential in the rapid correction of hyperglycemia and adjustment of antidiabetic therapy [107,110].

### 8.5. Preventive Measures

The prevention of PC-ONJ/CAM remains insufficiently standardized, but the literature draws attention to the fact that these entities derive from the accumulation of systemic and local risk factors, which can be influenced by integrated preventive measures [5,11].

#### 8.5.1. Use of Corticosteroid Therapy

Prolonged administration of corticosteroid therapy, at high doses or without a clear indication, increases the risk of CAM and maxillary osteonecrosis through immunosuppression, hyperglycemia, and endothelial dysfunction [107,110]. Moreover, it is recommended to limit corticosteroid therapy to absolutely necessary indications and carefully monitoring metabolic and infectious adverse effects [107,112].

#### 8.5.2. Metabolic Control and Diabetes

Several cases report an increased prevalence of diabetes, often uncontrolled, in patients who develop CAM/PC-ONJ, and Yasmin et al. explain the role of hyperglycemia in facilitating opportunistic infections and compromising healing. Therefore, the involvement of an endocrinologist and early optimization of glycemic control are presented as essential preventive measures [100,101,107,110].

#### 8.5.3. Dental Screening

Khan et al. and Al-Mahalawy et al. describe that seemingly minor dental procedures (e.g., extractions, endodontic treatments) can trigger necrosis in compromised vascular/inflammatory post-COVID, which is why the authors recommend dental evaluation and scanning of foci prior to invasive procedures in patients with a history of severe COVID-19 disease, corticosteroid therapy, and/or metabolic comorbidities [6,7].

#### 8.5.4. Post-COVID-19 Monitoring and Early Recognition

Initial symptoms, such as toothache, unexplained mobility, swelling may be nonspecific, leading to diagnostic delays; so that several authors recommend educating patients and primary care physicians to refer them promptly to specialized centers [5].

#### 8.5.5. Exposure Prevention and Environmental Control

Cornely et al. and Rudramurthy et al. note the importance of infection control, hygiene, and proper management of humidified oxygen therapy in hospital settings as measures that can reduce the risk of mucormycosis in vulnerable patients [105,106].

Overall, prevention is described as being multidimensional: therapeutic control of COVID-19, management of comorbidities, dental screening, and post-infectious monitoring. The correct implementation of these measures can reduce severe forms and improve the long-term prognosis of the disease.

### 8.6. Treatment Outcomes and Prognostic Factors

The assessment of therapeutic outcomes and prognostic factors is limited by clinical heterogeneity and the lack of standardized prospective studies, but case series and systematic reviews allow for the development of prognoses regarding mortality, cure, recurrence, and the role of the timing of surgical intervention [5,11].

#### 8.6.1. Mortality

Mortality in CAM remains significantly higher than in non-fungal PC-ONJ, with rates of 20–50% reported depending on extent, time of diagnosis, and presence of comorbidities [100,101,110]. The risk increases in orbital/cerebral extensions, especially when antifungal treatment and surgery are instituted late [100]. In contrast, predominantly non-fungal clinical cases report low mortality, often <5%, suggesting a more favorable prognosis in the absence of invasive infection [6,112].

#### 8.6.2. Healing and Local Interventions

In non-fungal PC-ONJ, sequesterectomy and local debridement are associated with healing/stabilization in most cases, with success rates exceeding 80% in some cases [7,112]. The authors note that sequestration delimitation and the absence of angioinvasion, favor therapeutic response [11]. In CAM, local control is more variable and depends on the extent and aggressiveness of surgery [101,118].

#### 8.6.3. Recurrence Rates

Recurrence is more relevant in CAM, where the literature frequently reports the need for repeated interventions due to subclinical progression or persistence of infected necrotic tissue [105,106]. Rudramurthy et al. highlight the association of recurrence with premature discontinuation of antifungals and insufficient metabolic control, especially in patients with uncontrolled diabetes mellitus [105]. Non-fungal PC-ONJ has a lower recurrence rate, usually related to the persistence of local or systemic risk factors [6].

#### 8.6.4. Surgical Timing and Prognosis

The timing of intervention is consistently described as a major determinant of prognosis. Early intervention, after metabolic stabilization but before massive extension, is associated with better survival and superior local control [5,100,101]. In CAM, postponing surgery in order to obtain an exclusively medical response is associated with rapid progression and poor prognosis [100]. In non-fungal PC-ONJ, a staged approach, with intervention when the sequestrum is well defined, can lead to excellent results [11].

#### 8.6.5. Major Prognostic Factors

Factors associated with poor prognosis include advanced age, uncontrolled diabetes, prolonged corticosteroid therapy, sinus/orbital extension, and delayed diagnosis. The prognosis is better in patients diagnosed early, with limited involvement, adequate metabolic control, and access to multidisciplinary care [5,11,100,110].

In conclusion, therapeutic outcomes depend on early recognition, correct differentiation between CAM and PC-ONJ, and prompt implementation of a strategy tailored to the severity and risk profile. Drug management in PC-ONJ differs fundamentally between invasive fungal disease and non-fungal PC-ONJ. Table 6 summarizes the proposed pharmacological management strategies for CAM-related osteomyelitis versus non-fungal PC-ONJ, highlighting indication-specific therapies, timing considerations, adjunctive treatments, and supportive metabolic measures, as derived from current international guidelines and published clinical series.

## 9. PC-RONJ Without Conventional Risk Factors

### 9.1. Aseptic PC-RONJ: Clinical Evidence in the Absence of Conventional Risk Factors

In recent years, the literature has begun to document a particular subgroup of patients who develop maxillary or mandibular osteonecrosis after SARS-CoV-2 infection, in the absence of conventional risk factors for osteonecrosis, such as antiresorptive or antiangiogenic treatment, head and neck radiotherapy, or histopathologically proven invasive mucormycosis. The authors used different terms to describe this phenomenon: PC-ONJ, PC-RONJ, or CRONJ, highlighting the lack of a clear nosological consensus [6,7,11].

However, the available clinical cases outline a relatively homogeneous clinical phenotype. Al-Mahalawy et al., in a multicenter review, reported predominantly maxillary involvement, with onset several weeks or months after recovery from COVID-19, under conditions of explicit exclusion of radiotherapy and antiresorptive therapy [7]. Similarly, Van Huynh et al. describe cases with no obvious trigger, i.e., no recent tooth extractions, local trauma, or other odontogenic procedures [108], a finding also reported by Grillo et al. in independent reports [85].

An essential element highlighted by several authors is the repeatedly negative results of fungal and bacterial investigations in these cases. Al-Mahalawy et al. and Sourani et al. mention negative fungal cultures and the absence of hyphae in PAS and GMS stains, arguing against a CAM etiology. Khan et al. also noted the lack of response to empirical antibiotic therapy, which led to a reassessment of the initial diagnosis of osteomyelitis [6,7,11].

Although the classic factors are absent, most patients have a particular history of COVID-19 disease: severe forms of SARS-CoV-2 infection, systemic corticosteroid therapy (usually dexamethasone), and diabetes mellitus or induced hyperglycemia, all of which are considered catalysts for endothelial dysfunction and bone ischemia [11,108]. The reported latency interval between SARS-CoV-2 infection and the onset of oro-maxillary symptoms ranged from a few weeks to 21 months, suggesting a subacute or delayed pathogenic mechanism [6].

An additional objective argument for an aseptic etiology is provided by Mañón et al., who documented focal arterial occlusion in the maxilla by CT angiography (CTA) in the absence of any microbiological evidence of infection. The authors interpreted the lesion as post-COVID-19 avascular necrosis, supporting the hypothesis of bone infarction secondary to SARS-CoV-2-associated coagulopathy [10].

Overall, the existing data indicate the existence of a real clinical subgroup of post-COVID-19 osteonecrosis occurring without conventional factors, which forms the basis for the nosological debate and the need for strict criteria to exclude infectious etiologies, aspects developed in the following subchapter [6,11].

### 9.2. Diagnostic Criteria, Rule-Out Strategy, and the Nosological Debate: Entity Versus Surrogate Label

One of the most controversial aspects of PC-RONJ is its differentiation from other established pathologies, such as chronic osteomyelitis, MRONJ, and ORN, as well as the rigorous exclusion of invasive fungal infections. The lack of standardized exclusion criteria has led to significant terminological overlap in the literature.

#### 9.2.1. Mandatory Rule-Out Criteria

The first diagnostic step is to exclude mucormycosis and other invasive fungal infections. Kang et al. and Khan et al. have demonstrated that the initial clinical presentation of CAM and non-fungal forms can be almost indistinguishable, requiring negative histopathological (PAS, GMS) and microbiological confirmation before invoking an aseptic mechanism [6,67]. Gaeta-Araujo et al. insist that the absence of fungal evidence must be explicitly documented, not assumed [109].

The second pillar is the exclusion of chronic bacterial osteomyelitis. Justine D. et al. point out that bone sequestration is not pathognomonic for osteonecrosis and may also be present in osteomyelitis [32]. In the well-documented PC-RONJ series, exclusion criteria included the absence of active pus, the absence of bacteria in the medullary spaces on special stains, and a minimal clinical response to isolated antibiotic therapy [11].

Exclusion of MRONJ requires clear documentation of the absence of exposure to antiresorptive or antiangiogenic agents. Yfanti et al. showed that imaging overlaps between MRONJ and PC-RONJ can be significant, which is why medication history remains the major defining criterion [8]. Similarly, ORN is excluded by the absence of a history of cervicofacial radiotherapy, an absolute negative criterion mentioned in all relevant series [7].

#### 9.2.2. Entity Versus Surrogate Label

After rigorous application of the exclusion criteria, the debate arises as to whether PC-RONJ is a distinct clinical entity or merely a surrogate label for atypical forms of multifactorial osteonecrosis. Khan et al. proposed the concept of “post-COVID-19 osteonecrosis masquerading as osteomyelitis,” suggesting that the primary process is ischemic, and that secondary inflammation generates diagnostic confusion [6].

In contrast, Romano et al. argue that the accumulation of cases without conventional factors, with spontaneous onset and prolonged latency, exceeds the threshold of a simple description and justifies the recognition of PC-RONJ as a distinct phenotype [104]. This position is methodologically supported by Yfanti et al., who identified imaging patterns suggestive of ischemic bone infarction with minimal peripheral inflammatory response [8].

Gaeta-Araujo et al. and Justine et al. conclude that PC-RONJ can be considered an emerging entity only conditionally, based on a documented and reproducible rule-out strategy, thus avoiding overdiagnosis [32,109].

### 9.3. Hypothesized Mechanisms: Microvascular Thrombosis and Bone Infarction as Primary Drivers

In the absence of a proven infectious etiology, the literature predominantly supports an ischemic-vascular mechanism for aseptic PC-RONJ [2,104,119]. SARS-CoV-2 is recognized as an agent with endothelial tropism, inducing systemic endotheliopathy characterized by endothelial inflammation, platelet activation, and disruption of anticoagulant mechanisms [2,119,120].

Becker et al. describe COVID-19-associated thrombosis as a distinct, immunologically mediated phenomenon in which the interaction between cytokines, the complement system, and neutrophil extracellular traps (NETs) promotes the formation of persistent microthrombi [121]. Conway et al. emphasize that this microangiopathy may persist post-acutely, explaining late ischemic complications [2].

The essential contribution of Mañón et al. lies in the direct imaging demonstration of maxillary arterial occlusion by CTA, in the absence of infection, providing a clear causal link between microthrombosis and bone necrosis. The authors showed that the distribution of the lesion corresponded to the affected vascular territory, supporting the concept of bone infarction [10].

There are also some particularities of maxillary circulation reported, such as terminal vascularization and intense bone remodeling, which may amplify vulnerability to repeated hypoperfusion. This model explains the spontaneous onset, prolonged latency, and lack of systemic signs of infection described in aseptic PC-RONJ [6,7,104].

### 9.4. Lessons from Case Reports: Clinical Behavior, Management, and Outcomes

Analysis of case reports highlights a clinical profile distinct from suppurative osteomyelitis and CAM [7,85,108]. A constant element is the onset without triggering factors, in the absence of dental procedures, an aspect highlighted and interpreted as an argument against a primary infectious etiology.

The evolution is described as slowly progressive, with gradual delimitation of the sequester, suggesting a chronic ischemic process [85,104]. Most authors report limited efficacy of antibiotic therapy in the absence of superinfection, while conservative sequestrectomy, performed after delimitation of the necrotic bone, is associated with healing or clinical stabilization without the need for extensive resections [7,108].

The prognosis is clearly more favorable compared to CAM, with no or very low mortality and low recurrence rates, conditional on the rigorous exclusion of fungal infections [85,108]. Moreover, the absence of microbial angioinvasion and relatively preserved immune status are positive prognostic factors [104].

In summary, these observations support the idea that aseptic PC-RONJ represents a distinct clinical phenotype, dominated by ischemic mechanisms, which requires a differentiated diagnostic and therapeutic approach. The defining clinical, etiological, and therapeutic features of aseptic PC-ONJ are summarized in Table 7.

## 10. Geographical and Ethnic Variations in Post-COVID-19 ONJ

**Figure 7:**
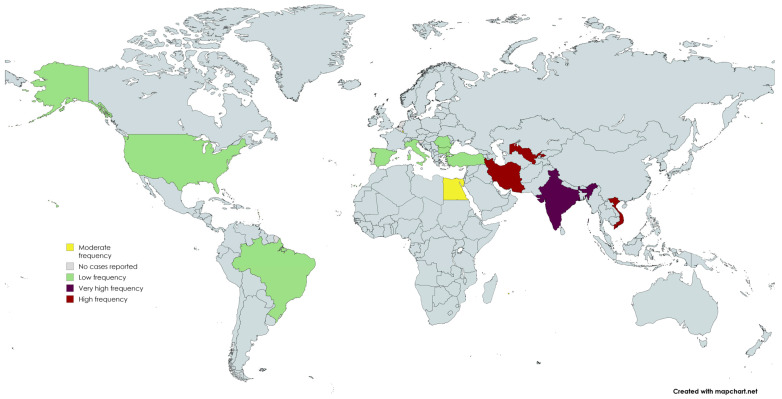
World map showing the frequency of reported cases of PC-ONJ. Color coding represents the relative frequency of reported cases of PC-ONJ or related maxillofacial complications in the scientific literature. The categories reflect the distribution of published reports rather than true epidemiological incidence. Countries shown in gray represent areas where no cases were identified in the reviewed literature at the time of analysis. Map template generated using MapChart (accessed 1 January 2026, https://www.mapchart.net/).

PC-ONJ demonstrates significant geographical variability in reported cases (Figure 7). The distribution of incidents across continents is not evenly distributed, which can be attributed to several factors: the severity of COVID-19, the therapeutic approaches that were used (particularly the use of corticosteroids), the prevalence of systemic comorbidities such as diabetes, environmental exposure to opportunistic pathogens, and the quality of healthcare systems. Furthermore, ethnic variations in thrombogenic and inflammatory responses may influence the susceptibility to post-COVID-19 vascular and bone complications. It is important to note that the majority of existing data are derived from case reports, small series, or regional outbreaks. Hence, the observed geographic patterns reflect differences in reported burden and phenotype, rather than the true incidence of the condition.

### 10.1. Continents

#### 10.1.1. Asia

India experienced the most significant outbreak of COVID-associated mucormycosis (CAM) globally, particularly during the second pandemic wave in mid-2021. During this period, tens of thousands of CAM cases were reported among COVID-19 patients. In many scenarios, it involved the rhino-sino-maxillary region. The outbreak was so severe that CAM was declared a notifiable disease in India on May 20, 2021, and was referred to as the “black fungus” epidemic. Many cases of CAM in India progressed to invasive rhino-maxillary mucormycosis with extensive necrosis of the maxilla. This geographic concentration and the maxilla-predominant phenotype of PC-ONJ in India distinguish it from patterns in other regions [122].

India has an estimated 77 million adults with diabetes (as of 2019) and an even larger number with prediabetes; approximately half are undiagnosed. Poor glycemic control was a central driver: in one analysis, 94% of COVID-associated mucormycosis patients had diabetes. Many CAM patients were only diagnosed with diabetes upon presentation. This high baseline of undetected or uncontrolled diabetes created a large susceptible population, as diabetic ketoacidosis and hyperglycemia promote fungal growth [123,124].

During the COVID-19 surges, systemic corticosteroids were used liberally, sometimes at excessive doses or durations, to treat patients, including those managed at home. This was identified as a key driver of the mucormycosis outbreak. In India, inappropriate or prolonged steroid regimens (often initiated early or given to mild cases) were common in the fearful pandemic context. Such misuse likely precipitated steroid-induced diabetes and further impaired immunity, directly increasing mucormycosis risk [123,124].

Hospital overload during COVID-19 surges contributed to delays in diagnosis and suboptimal infection control. Reports have indicated difficulties in maintaining sterile oxygen delivery setups. Reuse of humidifier water or non-sterile humidifiers, potentially increased exposure to environmental molds. One report noted that case overload led to improper disinfection of equipment, use of non-sterile or tap water in oxygen humidifiers was implicated as a risk for mucormycosis. The Indian Council of Medical Research responded by recommending only sterile water in humidifiers. Many patients were managed at home or in makeshift facilities where monitoring of steroid dosing and blood glucose was inconsistent. These conditions likely facilitated opportunistic fungal infections [125].

Also, during the pandemic, broad-spectrum antibiotics (most commonly azithromycin) were often given empirically or as prophylaxis to COVID-19 patients, which may have disrupted the normal oral and sinonasal microbiota that competitively inhibit fungal growth. Experts have cautioned that inappropriate blanket antibiotic use can lead to dysbiosis and reduced colonization resistance, thereby increasing CAM risk. In India, widespread availability of antibiotics without strict prescription control contributed to self-medication and overuse, which is considered an additional risk factor for CAM [125].

India’s climate (tropical and subtropical regions with hot, humid weather) and environment harbor abundant fungal spores. Warm, humid conditions favor the growth and proliferation of environmental *Mucorales* molds. In India, Rhizopus spores (the most common cause of mucormycosis) are ubiquitous in soil, air, and dust. The post-monsoon environment and ongoing construction activity can increase airborne spore loads. An analysis suggested that India’s climate combined with poorly maintained air conditioning/ventilation and non-sterilized reusable instruments may explain part of the CAM outbreak. Although *Mucorales* spores have been detected in Indian hospital air samples, their levels were generally low, suggesting the environment was a background factor rather than a sole cause [122].

Importantly, Asia has reported both infectious and non-infectious PC-ONJ phenotypes. While India’s cases were dominated by CAM-related maxillary osteonecrosis, there have also been cases of delayed jaw necrosis after COVID-19 in patients without any invasive fungal infection. An Indian case series described spontaneous maxillary osteonecrosis occurring weeks to months post-COVID-19 in patients who had received steroids but had no clinical or histological evidence of mucormycosis [7]. These non-fungal cases are hypothesized to result from COVID-19-related endothelial injury, hypercoagulability, and impaired bone healing, sometimes precipitated by a local insult like a dental extraction. In one Indian monocentric study (13 cases, including an 8-year-old child), PC-ONJ manifested as late as 21 months after COVID-19, often involving the maxilla (11 of 13 had maxillary involvement) with a history of corticosteroid use and, in many cases, a recent tooth extraction. About half of those patients were not diabetic, underscoring that even in the absence of traditional risk factors, the triad of COVID-associated coagulopathy, steroids, and a provoking dental event can lead to jaw osteonecrosis [6].

Neighboring South Asian countries and the Middle East also saw CAM cases during COVID-19 surges, though on a smaller scale than India. Iran, for example, reported cases of post-COVID-19 rhino-maxillary mucormycosis leading to jaw necrosis in severely ill diabetic patients. In one report from Sari, Iran, two uncontrolled diabetics developed maxillary osteonecrosis with “floating” teeth due to mucormycosis in the weeks following COVID-19. These cases underscore that in environments with similar risk profiles (diabetes prevalence, steroid use), COVID-19-related fungal osteonecrosis emerged outside India as well [66]. In another study from Iran, Grillo et al. reported two severe cases of CRONJ in Mashhad, one of which required extensive surgical intervention. Both Iranian patients had a history of serious COVID-19 illness, pointing to the need for maxillofacial surgeons to be vigilant for jaw necrosis in recovered patients [85].

Further north in Central Asia, an unusually large cluster of post-COVID-19 jaw osteomyelitis was observed in Uzbekistan. There were described 52 cases of patients who developed chronic osteomyelitis of the jaws within 6–8 months after COVID-19, often with slow, progressive necrosis and delayed sequestra formation. Many of these Uzbekistani patients suffered complications like cavernous sinus thrombosis, orbital cellulitis, and facial vein thrombophlebitis due to the extensive necrotic infection, underscoring the severe end of the spectrum of PC-ONJ in that region. Environmental factors, such as poorly controlled diabetes and exposure to fungal spores, likely contributed to these aggressive presentations [97].

Fewer cases have been reported in the Far East, but a marked series comes from Vietnam. Huynh et al. prospectively studied nine patients in Ho Chi Minh City who developed post-COVID-19 jaw osteonecrosis. All Vietnamese patients had maxillary involvement and were diabetic; each had received corticosteroids during COVID-19 management. The average interval from COVID-19 recovery to jaw symptom onset was about 4–5 weeks. Clinically, they presented with mobile maxillary segments, exposed necrotic bone, and sinus tract formation similar to cases elsewhere. All were treated with surgical debridement, resulting in satisfactory healing [108].

#### 10.1.2. Europe

In Europe, PC-ONJ appears to be rare, documented primarily through isolated case reports rather than outbreaks. No large clusters similar to South Asia’s epidemic have been reported. The few published European cases illustrate that jaw osteonecrosis can occur after COVID-19 even in patients with no history of antiresorptive medications or head-and-neck radiation (ruling out medication-related or radiation-induced ONJ). A case in Eastern Europe described post-COVID-19 maxillary osteonecrosis in the absence of any fungal infection, supporting an ischemic or inflammatory pathogenesis. The patient developed chronic necrosis of the maxilla several weeks after recovering from COVID-19 and corticosteroid treatment, and an extensive workup found no evidence of mucormycosis or bacterial osteomyelitis. Such cases highlight a diagnostic challenge: PC-ONJ in Europe may mimic chronic osteomyelitis or even malignancy, and careful exclusion of other etiologies is required [126].

Another representative European case comes from Bulgaria. Slavkova and Nedevska reported an aseptic necrosis of the maxilla in a 70-year-old male approximately two months after a severe COVID-19 infection. The patient had no known comorbidities but had endured a protracted ICU stay with intensive COVID-19 treatments. He developed swelling and fistulae in the maxillary region soon after hospital discharge. Imaging showed a “moth-eaten” destruction of the maxillary bone, and the diagnosis of aseptic osteonecrosis of the maxilla was made after biopsies revealed necrotic bone without invasive fungus. The case was managed with surgical resection of the necrotic bone. This Bulgarian report is notable because it demonstrated, via CT angiography, an occlusion in the maxillary arterial supply associated with the necrotic segment. It underscores that even in the absence of infection, COVID-induced coagulopathy can lead to localized infarction of the jaw [127]. Cases from Italy have described maxillary osteonecrosis developing weeks to months after severe COVID-19 infection, frequently in association with systemic corticosteroid therapy, endothelial dysfunction, and hypercoagulability, but without consistent evidence of invasive fungal disease [104]. Similarly, a case report from Turkey has demonstrated maxillary-predominant osteonecrosis following COVID-19, including rare presentations complicated by secondary bacterial infections such as actinomycosis. These cases are notable for occurring even in patients without classic systemic comorbidities, highlighting the potential role of COVID-19-related immune dysregulation, microvascular injury, and prolonged corticosteroid exposure as sufficient triggers [102].

#### 10.1.3. America

North America has reported very few PC-ONJ cases, mostly isolated reports in the literature. A notable example in the United States was published by Mañón et al., describing a COVID-associated avascular necrosis of the maxilla. In this case, a 72-year-old male in Texas developed an infarction of the maxillary bone as a sequela of severe COVID-19 illness. There was no evidence of fungal infection; instead, the necrosis was attributed to COVID-induced hypercoagulability and endothelial dysfunction, which likely compromised the blood supply to the maxilla. The patient required surgical debridement of the devitalized bone. The authors emphasized that COVID-19 confers a prothrombotic state leading to various thrombo-ischemic complications, and suggested that aggressive thromboembolic prophylaxis during and after COVID-19 could be important to prevent such outcomes [10].

Overall, the North American experience suggests that PC-ONJ is a rare complication of COVID-19. Unlike in South Asia, there was no surge of cases across the continent. COVID-associated mucormycosis did occur in North America but remained uncommon, typically confined to patients with severe diabetes or profound immunosuppression in isolated incidents. There was no indication of any widespread outbreak of CAM or spike in jaw necrosis post-COVID-19 in the general population. This relative rarity in North America is likely due to tighter metabolic control (better baseline diabetes management), early access to medical care (preventing minor infections from becoming severe), and standardized steroid protocols (reducing inappropriate corticosteroid exposure). In summary, while PC-ONJ can occur in North America, it has so far been an uncommon, idiosyncratic event rather than a recognized public health pattern [10].

Similarly, in South America, PC-ONJ is scarcely documented. In Brazil, Riva et al. described a case of a 61-year-old patient who presented with acute osteonecrosis aggravated by SARS-CoV-2. The patient’s infection was aggravated by the formation of microthrombi due to SARS-CoV-2. The patient also had diabetes mellitus and was undergoing treatment with corticosteroids. In this case, a multifactorial pathogenesis is suggested, in which SARS-CoV-2 infection may have acted as a triggering or potentiating factor rather than the sole cause of jaw osteonecrosis [128].

#### 10.1.4. Africa

Data of PC-ONJ in Africa are scarce, with Egyptian data being the most prevalent. Al-Mahalawy and colleagues presented a retrospective analysis of 12 individuals who experienced maxillary osteonecrosis subsequent to recovering from SARS-CoV-2 infection. All subjects had systemic risk factors, predominantly diabetes mellitus, and received corticosteroid treatment during their COVID-19 management. The average duration between recovery and the manifestation of symptoms was roughly 5.5 weeks. Clinically, the cases were distinguished by exposed necrotic maxillary bone, purulent discharge, and dentoalveolar mobility. Microbiological and histopathological examinations revealed no evidence of fungal infection, thereby differentiating these instances from the jaw necrosis associated with COVID-19 in South Asia. The authors suggested a multifactorial origin, encompassing COVID-19-induced endothelial dysfunction, hypercoagulability, corticosteroid administration, and metabolic disturbances. While these cases are described as a significant complication, they do not indicate a widespread outbreak in Africa [7].

### 10.2. Ethnic Variations

Beyond geography, inter-individual variation in thrombo-inflammatory biology may plausibly modulate susceptibility to post-COVID-19 vascular complications, including ischemic injury to bone. Population-level studies in cardiovascular and hematologic literature show that average baseline levels of some coagulation and endothelial markers (e.g., factor VIII and von Willebrand factor) differ across ancestry groups. For example, higher circulating levels of factor VIII and von Willebrand factor have been reported on average in African ancestry populations compared with White European ancestry populations, whereas Asian ancestry populations tend to show lower rates of venous thromboembolism in many non-COVID-19 settings [129]. In parallel, reviews of antithrombotic response describe the so-called “East Asian paradox”, reflecting, on average, lower thrombotic propensity and/or different hemostatic balance with higher bleeding liability under antithrombotic therapies in East Asian populations [129,130].

However, no study has directly demonstrated an association between ethnicity and PC-ONJ incidence. Any proposed link, therefore, remains biologically plausible but unproven and should be interpreted cautiously. First, the clinical entity of PC-ONJ itself is heterogeneous (infectious CAM-associated necrosis versus apparently non-fungal ischemic/inflammatory necrosis), and most published evidence consists of case reports or small series. Second, ancestry-associated averages are small compared with the effects of major drivers that clearly cluster PC-ONJ risk: uncontrolled diabetes, systemic corticosteroid exposure (dose, timing, duration), severity of COVID-19, and triggering local factors such as dental extraction [6,131]. Third, ethnicity is a social and biological construct that does not map cleanly to genetics. Within-group heterogeneity is substantial, and socioeconomic and healthcare determinants often dominate observed disparities.

Importantly, the scarcity of published PC-ONJ from Africa should not be interpreted as evidence of low biological susceptibility. Reviews of mucormycosis epidemiology in Africa emphasize that case counts are strongly influenced by diagnostic capacity and reporting. The disease is rarely reported in many sub-Saharan settings despite likely under-recognition, and authors have called for registries and standardized surveillance to clarify the true burden [132]. Thus, while thrombo-inflammatory differences across populations may contribute to risk modulation, the current geographic distribution of PC-ONJ publications is more plausibly explained by differences in exposure profiles (diabetes, steroid practices, environmental fungal exposure) and health-system factors (recognition, mycologic workup, referral patterns, and publication output) than by ancestry alone.

Ancestry-linked thrombotic and inflammatory biology may influence vulnerability to post-COVID-19 microvascular injury, but PC-ONJ remains a context-dependent complication in which comorbidities, treatment exposures, and healthcare systems are the dominant determinants; ethnicity/ancestry should be framed as a potential modifier, not a primary explanation [129].

Overall, PC-ONJ exhibits marked global variability in reported cases and phenotypes, but the present literature cannot define true incidence because it is dominated by case reports, small series, and regional clusters. Asia, particularly India, stands out for a CAM-driven, maxilla-predominant phenotype that aligns with convergent risk factors (high diabetes burden, widespread corticosteroid exposure during surges, and environmental exposure to *Mucorales*), whereas Europe and North America have largely reported sporadic, non-fungal cases consistent with ischemic and thrombo-inflammatory mechanisms.

Ethnicity-linked differences in coagulation and inflammatory profiles may act as biologic modifiers of post-COVID-19 vascular injury, but they do not currently explain geographic reporting patterns, and they should not be interpreted independently of comorbidities and healthcare context. Finally, limited reporting from some regions, particularly parts of Africa, may reflect under-recognition, diagnostic constraints, and publication bias rather than the absence of disease. Future progress requires multicenter registries with standardized diagnostic criteria (including systematic exclusion of fungal disease when relevant), consistent reporting of steroid exposure and metabolic status, and longitudinal follow-up to distinguish infectious CAM-associated osteonecrosis from delayed, non-infectious post-COVID-19 jaw infarction.

## 11. Comparison with Other ONJ Etiologies

Comparative analysis of maxillary osteonecrosis occurring in the context of PC-ONJ with that of classic maxillary osteonecrosis pathologies is essential for clarifying its nosological position and avoiding diagnostic and management errors. Recent studies and case reports point out that, although PC-ONJ may present clinical and imaging features also found in other established pathologies, it does not completely overlap with any of them: the differences stem from the patient’s history, the pathogenic mechanism, and the clinical evolution, which are significant [6,7,11,104].

### 11.1. Versus MRONJ

Authors describing MRONJ consistently emphasize that the central element of this entity is the patients’ medication history, particularly exposure to antiresorptive agents (bisphosphonates, denosumab) and/or antiangiogenic agents for cumulative periods of months or years [133,134,135].

According to the AAOMS and MASCC/ISOO positions, the clinical definition of MRONJ includes the presence of exposed or palpable intraoral bone that does not heal for at least 8 weeks in patients with documented drug exposure and no history of cervico-facial radiotherapy [16]. From an epidemiological point of view, the authors report major differences in risk depending on the therapeutic indication: in osteoporosis, the risk is estimated at 0.02–0.05% for bisphosphonates and 0.04–0.3% for denosumab, while in the oncological population the cumulative risk can reach up to 8%, with reported ranges for zoledronate between 0 and 18% and for denosumab between 0 and 6.9%, with most studies focusing below the 5% threshold [16,134,136].

Regarding local triggering factors, several authors highlight that tooth extractions precede the onset of MRONJ in up to 82% of cases, and the anatomical distribution is predominantly mandibular (approximately 75% mandible vs. 25% maxilla) [134].

In contrast, case studies dedicated to PC-ONJ explicitly exclude any history of antiresorptive or antiangiogenic therapy, and the clinical onset is reported as acute or subacute, weeks or months after SARS-CoV-2 infection, not after chronic drug exposure [6,7,11]. The authors highlight that this temporal difference is one of the strongest arguments for correctly classifying the pathology as PC-ONJ and not diagnosing MRONJ.

The clinical course also differs significantly. MRONJ is characterized by a slow progressive evolution, often linked to a triggering factor such as dental procedures, while PC-ONJ is frequently described as having a “spontaneous” onset, without an identifiable dental trigger factor, an aspect reported in more than half of non-fungal cases [6,7].

Therapeutic protocols reflect these pathogenic differences. In MRONJ, international guidelines initially recommend prolonged conservative management with antibiotic therapy and oral antiseptics, with surgical interventions reserved for advanced stages [133,135]. In contrast, in PC-ONJ, several authors report favorable outcomes after early conservative sequestrectomy, once the delimitation of necrotic bone is clear, suggesting a pathophysiology closer to bone infarction than to chronically suppressed remodeling [7,11].

### 11.2. Versus ORN

ORN is unanimously recognized as a late complication of head and neck radiotherapy, being determined by hypocellularity, hypovascularization, and progressive tissue hypoxia in a well-defined irradiated area [137,138].

The authors indicate that the severity of ORN is closely dependent on the total radiation dose, fractionation, and exposed bone volume, and the latency interval may exceed 12–24 months after completion of radiotherapy. From a therapeutic point of view, approaches range from irrigation, debridement, and antibiotic therapy to adjuvant medical regimens, such as pentoxifylline 400 mg twice daily combined with vitamin E 500 IU twice daily for a minimum of 6 months, and, in severe forms, extensive bone resections with free flap reconstruction [138].

In contrast, all PC-ONJ clinical cases analyzed explicitly exclude a history of cervicofacial radiotherapy, and the distribution of lesions does not follow an irradiated field but rather a vascular territory, suggesting a different mechanism [11,104].

Although both ORN and PC-ONJ may share clinical features such as exposed bone, sequestration, and difficult healing, the underlying mechanisms differ fundamentally. Romano et al. draw attention that ischemia in ORN is the consequence of chronic radiation-induced vascular injury, while in PC-ONJ, ischemia is attributed to COVID-19-associated endotheliopathy and post-viral microthrombosis [104,119].

In terms of management, there are partial overlaps, such as the use of conservative debridement and hyperbaric oxygen therapy as adjuvants. However, the cure rate with conservative treatment alone in ORN is low (approximately 15%), frequently requiring escalation to surgical interventions, while in aseptic PC-ONJ, conservative surgery is often sufficient [11,138].

### 11.3. Overlap with Chronic Osteomyelitis of the Jaw

The clinical overlap between PC-ONJ and chronic osteomyelitis of the jaws (OM) is one of the main sources of diagnostic confusion. Several authors warn that pain, exposed bone, sequestration, and fistulas can occur in both pathologies [32,139,140].

However, the literature on chronic osteomyelitis underlines that it is almost invariably associated with an odontogenic or traumatic focus, bacterial isolation, and a response, even partial, to prolonged antibiotic therapy [140,141].

In contrast, Khan et al. and Vardas et al. describe that many cases initially labeled as post-COVID-19 osteomyelitis do not show active pus, positive bacterial cultures, or clinical response to antibiotic therapy alone, and histology predominantly shows necrotic bone with minimal secondary inflammation. These observations support the hypothesis that inflammation is a secondary phenomenon to ischemia, not the primary mechanism [5,6].

An important clinical clue is the post-COVID-19 latency, reported to be up to 21 months in some cases, which is unusual for classic odontogenic osteomyelitis [6]. Also, the predominantly maxillary involvement, reported in over 80–90% of PC-ONJ cases, contrasts with the typical distribution of osteomyelitis, which more commonly affects the mandible [5].

Justine et al. emphasize that the presence of bone sequestration is not pathognomonic for osteomyelitis, and the differentiating criterion remains the etiological context and biological behavior of the lesion [32].

### 11.4. Implications for Classification

From the perspective of existing classifications, the authors underline that MRONJ systems are built around drug exposure and the 8-week time criterion, which makes it difficult to directly include PC-ONJ [133,134].

Similarly, ORN classifications are based on the severity and extent of lesions in relation to the irradiated field, a criterion absent in PC-ONJ [137,138].

Sourani et al. point out that the interchangeable use of the terms osteomyelitis and osteonecrosis in the post-COVID-19 context has contributed to the current nosological confusion [11]. Romano et al. and Khan et al. argue that the accumulation of cases without conventional factors, with post-viral onset, a plausible vascular mechanism, and distinct clinical behavior, warrants the recognition of PC-ONJ as an emerging clinical phenotype, subject to strict exclusion criteria [6,104].

In contrast, Vardas et al. take a more cautious stance, suggesting that PC-ONJ may represent a spectrum of osteonecrosis precipitated by COVID-19, but not yet a separate entity, emphasizing the need for prospective studies and consensus criteria [5].

The current literature suggests that PC-ONJ should either be introduced as a distinct subcategory in ONJ classifications or explicitly mentioned as osteonecrosis associated with SARS-CoV-2 infection, with a rigorous differential diagnosis algorithm.

## 12. Future Directions and Research Gaps

Despite the rapidly expanding number of case reports and small series, PC-ONJ remains an incompletely defined, heterogeneous clinical spectrum rather than a single, uniformly characterized disease entity. Current evidence is dominated by retrospective observations and publication-driven clustering, which limits inference regarding true incidence, causality, and optimal management. Systematic reviews confirm that the available literature is composed largely of low-level evidence (case reports/series), with substantial variability in diagnostic work-up, terminology, and follow-up reporting [5,11].

A central gap is the lack of standardized diagnostic criteria that can reproducibly separate (i) CAM-related invasive fungal osteomyelitis/osteonecrosis, (ii) bacterial osteomyelitis phenotypes, and (iii) aseptic, ischemic PC-RONJ-like forms. Without harmonized rule-out criteria, the same phenotype is variably labeled “osteomyelitis,” “osteonecrosis,” “avascular necrosis”, or “PC-RONJ”, perpetuating nosological drift and limiting comparability across cohorts [5,7,11].

Future work should prioritize an international consensus statement that defines minimum diagnostic requirements (histopathology, fungal-directed microbiology, and standardized reporting of medication/radiation exposure), and explicitly frames PC-ONJ as an etiologically stratified umbrella (CAM vs. non-fungal ischemic/inflammatory vs. bacterial osteomyelitis-dominant) [11].

Another major gap is the absence of population denominators. Current reviews summarize reported cases, but cannot estimate incidence, because case ascertainment is driven by referral patterns, diagnostic capacity, and regional outbreaks (especially CAM) [5].

Multicenter prospective registries are needed to capture consecutive patients with standardized diagnostic workflows, enabling valid incidence estimates, phenotypic clustering, and outcome benchmarking. Such registries should also document COVID-19 severity, hospitalization, oxygen/ICU exposures, timing and cumulative steroid dosing, glycemic trajectories (including steroid-induced hyperglycemia), and local triggers (extractions, dentures, periodontal status) [5].

Mechanistically, the current manuscript correctly frames PC-ONJ as multifactorial, but the relative contribution of microthrombosis/endotheliopathy versus secondary infection remains poorly quantified, particularly in “aseptic” cohorts. A key research direction is to move from plausibility models to measurable correlates: vascular imaging (CTA/MRA), perfusion assessments, and histopathologic scoring of thrombosis/endarteritis patterns, aligned with coagulation and endothelial biomarkers (e.g., vWF, factor VIII, D-dimer trajectories) where feasible [5,7].

Equally important is microbiologic granularity. Studies should distinguish colonization from invasive disease using tissue-based criteria (angioinvasion) and standardized sampling (deep tissue, not superficial swabs), because misclassification in either direction (overcalling CAM or undercalling CAM) has direct therapeutic consequences [106].

CT/CBCT and MRI are indispensable for extension and severity assessment yet remain limited for etiologic discrimination in ambiguous cases. The research gap is not whether imaging detects disease, but whether imaging can support probabilistic triage (urgent CAM pathway vs. staged ischemic/sequestration pathway) before tissue confirmation. Prospective radiology-pathology correlation studies are needed to evaluate whether patterns such as mucosal non-enhancement, “black turbinate” surrogates, marrow signal signatures, or territorial infarction-like distributions improve pre-biopsy risk stratification [11].

Future studies should (i) standardize outcome definitions (healing, recurrence, fistula closure, functional rehabilitation), (ii) compare early conservative surgery (timed sequestrectomy) versus prolonged conservative measures in non-fungal phenotypes, and (iii) evaluate adjuvants (HBOT, PRP, photobiomodulation) using controlled designs rather than isolated case-based claims [11].

A practical research priority is the development of post-COVID-19 dental/OMFS risk stratification tools that integrate systemic risk (diabetes control, steroid exposure, immunomodulators), local risk (periodontitis, planned extractions), and temporal proximity to SARS-CoV-2 infection. This is particularly relevant because systematic reviews identify diabetes and corticosteroid exposure as dominant recurring features, but thresholds (dose, duration, timing) remain imprecise [5,7].

Prospective protocols should test whether structured screening (glycemic optimization, delayed elective extractions in high-risk windows, early imaging escalation for disproportionate pain/mobility) reduces late diagnosis and prevents progression to extensive defects.

Finally, geographic variability in published PC-ONJ likely reflects exposure profiles (diabetes burden, steroid practices, CAM outbreaks) and diagnostic capacity rather than intrinsic susceptibility alone. A key gap is the lack of standardized surveillance across regions, particularly in regions where access to fungal diagnostics and histopathology is limited. Future multicountry collaborations and minimal datasets would reduce publication bias and allow genuine comparisons of phenotype distributions (CAM-dominant vs. non-fungal ischemic phenotypes) across health systems [5].

In summary, the next phase of PC-ONJ research should aim to stabilize terminology via consensus case definitions, build prospective registries with denominators and standardized rule-out pathways, quantify mechanistic signatures of ischemia versus invasion, and generate comparative effectiveness evidence for staged surgery, antimicrobial stewardship, and adjuvant therapies, so that “post-COVID-19 context” becomes a structured risk framework rather than a residual label [5]. This review offers a novel contribution by presenting a comprehensive, etiology-driven framework for understanding post-COVID-19 jaw osteonecrosis. Instead of considering PC-ONJ as a singular condition, this paper offers a critical synthesis of existing research across infectious (CAM-related), non-fungal ischemic, and mixed phenotypes. It addresses diagnostic challenges, nosological uncertainties, and the overlapping clinical features. Furthermore, this paper seeks to bridge current research deficiencies by synthesizing epidemiological data, pathophysiological processes, imaging results, histopathological features, and treatment strategies into a unified clinical framework, highlighting the importance of rigorous exclusion criteria and collaborative assessment.

## 13. Conclusions

ONJ occurring after SARS-CoV-2 infection represents a heterogeneous post-COVID-19 complication, rather than a single, clearly defined disease entity. The available evidence supports a spectrum of post-COVID-19 jaw pathologies, ranging from aggressive CAM to non-fungal, predominantly ischemic forms of jaw osteonecrosis, each with distinct mechanisms, prognostic implications, and therapeutic requirements.

COVID-19 appears to act primarily as a systemic risk amplifier, through endothelial dysfunction, microvascular thrombosis, immune dysregulation, metabolic imbalance, and treatment-related factors, most notably systemic corticosteroid exposure, rather than as a direct cause of jaw necrosis. The predominance of maxillary involvement, variable latency after infection, and frequent absence of early bone exposure contribute to significant diagnostic challenges and explain the frequent overlap with chronic osteomyelitis, MRONJ, and ORN.

Accurate diagnosis relies on etiology-driven assessment, in which imaging defines disease extent but cannot determine causation. Therefore, accurately excluding invasive fungal infections and other known causes through histopathology and targeted microbiology is essential to avoid both undertreatment and overtreatment. Successful treatment depends on early diagnosis, correct classification, and coordinated care from different medical specialties.

Ultimately, PC-ONJ should be seen as a complex condition with multiple causes, requiring careful categorization. To improve clinical decisions, refine treatment plans, and better understand the true impact and causes of this new post-viral condition, it is crucial to establish standardized diagnostic criteria, agree on common terminology, and collect data from multiple centers in a planned way.

## Figures and Tables

**Table 2:** Clinical and extension indicators with practical value in differentiating post-COVID-19 phenotypes of maxillary osteonecrosis.

Clinical/Paraclinical Indicator	Practical Significance	Reference
Variable post-COVID-19 latency (average ~100 days; wide range up to >600 days)	Supports the subacute or late nature of post-COVID-19 forms; late onset does not preclude severity	[5]
Predominance of maxillary involvement (>90% of cases)	Atypical pattern compared to classic odontogenic osteomyelitis; suggests sinonasal or ischemic component	[5]
Frequent sinus involvement (~60%)	Indirect marker of loco-regional extension and potential severity	[5]
Tooth pain and mobility before bone exposure	Lesion may be symptomatic from onset, even in the absence of exposed bone	[5]
Onset after extraction with palatal swelling, sequestration, and exposed bone	Suggests an osteomyelitis-like phenotype precipitated by the procedure	[6]
Oro-antral/oro-nasal communication requiring an obturator	Indicator of functional severity and sinus extension	[101]
Palatal discoloration, palatal erosion, sinus drainage	Oral signs that may indicate rhinorectal extension	[101]
Distribution of severity in CAM (35% sinus, 50% rhinorectal, 15% rhinorectal-cerebral)	Guidance framework for risk stratification in invasive forms	[101]
“Black turbinate sign” and orbital/intracranial signs	Suggest fungal invasion; requires urgent imaging and tissue confirmation	[103]

**Table 3:** Characterization of patient profile [5].

Parameter	Reported Result
Gender	71.7% men; 28.3% women
Age	Median 52.72 years; range 8–83
Diabetes	65.1%
Hypertension	19.8%
No reported comorbidities	5.7%
Corticosteroid therapy in COVID-19	58.5%
Hospitalization for COVID-19	41.5%

**Table 4:** Diagnostic workflow for suspected PC-ONJ.

Diagnostic Step	Key Clinical/Paraclinical Elements	Main Diagnostic Pitfalls	Role in Diagnostic Algorithm	References
**1. Clinical presentation**	History of SARS-CoV-2 infection (weeks–months prior) with persistent oro-facial or jaw pain, facial swelling, sudden tooth mobility, exposed bone or non-healing extraction site, purulent or fetid discharge, oro-antral/oro-nasal fistula	Misinterpretation as routine odontogenic infection or uncomplicated osteomyelitis	Triggers suspicion of PC-ONJ or CAM-related disease	[6,11,100]
**2. Initial clinical risk stratification**	Rapid symptom progression, pain disproportionate to findings, neurological signs (paresthesia/anesthesia), sinonasal symptoms, uncontrolled diabetes, recent or high-dose corticosteroid therapy	Underestimation of severity in absence of early bone exposure	Identifies high-risk presentations and indicates need for expedited work-up	[5,67,100]
**3. First-line imaging**	OPG and/or CT showing osteolytic lesions, sequestrum formation, loss of trabecular architecture, cortical bone destruction	Imaging overlap with MRONJ, ORN, chronic osteomyelitis, malignancy	Confirms bone involvement and delineates extent, but does not establish etiology	[5,32]
**4. Advanced imaging**	MRI findings: non-enhancing ischemic or necrotic mucosa, bone marrow signal alterations, soft-tissue, orbital or vascular extension	Confusion with inflammatory sinus disease or neoplasia	Refines assessment of severity and extension; cannot replace tissue diagnosis	[103]
**5. Etiologic suspicion node**	Rapidly progressive course, early sinonasal/orbital involvement, ischemic MRI patterns, poor or absent response to empirical antibiotics	Delayed recognition of invasive fungal disease	Determines need for urgent tissue-based diagnosis	[105,107]
**6. Tissue-based diagnosis (mandatory in ambiguous cases)**	Histopathology for fungal hyphae and angioinvasion; fungal ± bacterial cultures (when possible); exclusion of malignancy	False-negative results due to superficial or inadequate biopsy	Gold standard for confirming CAM and excluding alternative diagnoses	[11,106,107]
**7. Diagnostic classification**	A. CAM-related invasive fungal osteomyelitis: positive histopathology → urgent antifungal + surgical management; B. Non-fungal PC-ONJ: negative fungal studies, ischemic necrosis/sequestration → debridement ± conservative care; C. Alternative diagnosis: osteomyelitis, MRONJ, malignancy	Over-diagnosis of CAM or under-diagnosis of invasive disease	Directs etiology specific therapeutic strategy	[6,7,100,108]
**8. Multidisciplinary validation**	Joint evaluation by OMFS, ENT, radiology, infectious diseases/pathology	Fragmented, single-discipline assessment	Minimizes misclassification, reduces diagnostic delay, aligns treatment strategy	[5,105]

**Table 5:** Proposed diagnostic algorithm in PCONJ and CAM-ROCM.

Diagnostic Stage	Essential Clinical/Paraclinical Elements	Common Diagnostic Errors	Practical Role in the Algorithm	Sources
**Initial clinical presentation**	Persistent orofacial pain, swelling, sudden tooth mobility, exposed/probing bone, oroantral/oronasal fistulas, halitosis	Attribution of symptoms to a common dental infection or chronic osteomyelitis	Starting point in the algorithm; raises suspicion of PCONJ/CAM in a post-COVID-19 context	[5,6,11]
**Identification of risk factors**	Rapid onset, accelerated progression, disproportionate pain, paresthesia, sinonasal/orbital symptoms, diabetes mellitus, corticosteroid therapy	Underestimation of severity in the absence of initial bone exposure	Risk stratification: urgency of investigations and need for biopsy to be decided	[5,67,100]
**Initial imaging (OPG)**	Osteolysis, loss of trabecular architecture, bone sequestration	Initial OPG not suggestive → false sense of benignity	Initial screening; does NOT rule out active disease	[32,67]
**Extension imaging (CT/CBCT)**	Cortical destruction, sequestration, relationship with maxillary sinus, sinus erosions	CT interpretation as etiological test	Anatomical mapping (bone + sinus); does NOT establish etiology	[5,7,32]
**Advanced imaging (MRI)**	Medullary edema, areas of contrast non-enhancement, extension into soft tissues/orbit	Confusion with inflammatory sinusitis or neoplasia	Assesses severity and extent; does NOT replace biopsy	[32,103]
**Suspicion of CAM/ROCM**	Rapid progression, sinonasal/orbital involvement, lack of response to antibiotics	Late initiation of antifungal therapy or based solely on CT	Triggers urgent biopsy and multidisciplinary evaluation	[105,107]
**Biopsy + histopathology**	Fungal hyphae, angioinvasion, thrombosis, ischemic necrosis	False negative if biopsy is superficial or from necrotic tissue	Gold standard for CAM; excludes malignancy	[11,105,106]
**Targeted microbiology**	Fungal (*Mucorales*/*Aspergillus*) + bacterial cultures; PCR from fresh tissue	Negative cultures ≠ exclusion of invasive infection	Complements diagnosis; guides therapy, does not decide alone	[104,105,106]
**Active differential diagnosis**	Chronic osteomyelitis, MRONJ, ORN, malignancies	Premature labeling as “simple osteomyelitis”	Avoids inappropriate treatments and critical delays	[6,32,109]
**Interdisciplinary integration (OMFS–ENT–Radiology–ID)**	Clinical + imaging + tissue correlation	Fragmented, single-discipline approach	Reduces classification errors; optimizes therapeutic timing	[5,105]
**Final case classification**	Confirmed CAM vs. non-fungal PCONJ vs. other etiology	Overdiagnosis of CAM or underdiagnosis of invasive forms	Directs the correct therapeutic approach	[7,100,108]

Abbreviations: OPG = orthopantomogram; CBCT = cone-beam computed tomography; CT = computed tomography; MRI = magnetic resonance imaging; PCR = polymerase chain reaction; ENT = ear, nose, and throat; OMFS = oral and maxillofacial surgery.

**Table 6:** Proposed drug management: CAM (fungal) vs. PC-ONJ (non-fungal).

Element (Medication)	CAM (Fungal)	PC-ONJ (Non-Fungal)	References
**Specific therapy**	L-AmB 5–10 mg/kg/day (first line).	Antifungals are not recommended without histopathological/microbiological confirmation.	[7,16,106,107]
**Antifungal timing**	Delay ≥6 days associated with major increase in mortality (from 49% to 83% at 12 weeks).		[7,110]
**Step-down/rescue**	Isavuconazole or posaconazole (step-down/rescue, including for renal toxicity/CI to AmB).		[16,105,106,110]
**Antibiotics**	Only if bacterial superinfection is suspected/confirmed (as part of a multimodal approach).	Only in the presence of signs of bacterial superinfection (stewardship).	[7,16,100,105]
**Oral antiseptics**	Adjuvant in multimodal protocols.	Standard: 0.12–0.2% chlorhexidine in conservative/postoperative treatment.	[11,16,100]
**Metabolic support (associated medication)**	Rapid/strict glycemic control (impact on response, recurrence).	Metabolic control recommended as a baseline measure.	[5,7,105,107,110]

Abbreviations: CAM = COVID-19-associated mucormycosis; PC-ONJ = post-COVID-19 jaw osteonecrosis; L-AmB = liposomal amphotericin B; AmB = amphotericin B.

**Table 7:** Key characteristics of aseptic PC-RONJ.

Characteristics	Main Observations	References
Conventional factors	Absent (no antiresorptives, radiotherapy, CAM)	[7,11]
Onset	Spontaneous, no trigger	[85,108]
Fungal investigations	Negative (PAS/GMS/cultures)	[6,7]
Dominant mechanism	Ischemic (microthrombosis, bone infarction)	[10,119]
Effective treatment	Conservative sequestrectomy	[108]
Prognosis	Favorable, minimal mortality	[104]

Abbreviations: PAS = periodic acid–Schiff; GMS = Grocott methenamine silver; CAM = COVID-19-associated mucormycosis.

## Data Availability

The original contributions presented in this study are included in the article. Further inquiries can be directed to the corresponding authors.
